# Anxiety Disorders in Williams Syndrome Contrasted with Intellectual Disability and the General Population: A Systematic Review and Meta-Analysis

**DOI:** 10.1007/s10803-016-2909-z

**Published:** 2016-09-30

**Authors:** R. Royston, P. Howlin, J. Waite, C. Oliver

**Affiliations:** 1 The Cerebra Centre for Neurodevelopmental Disorders, School of Psychology, University of Birmingham, Edgbaston, Birmingham, B15 2TT UK; 2 Department of Psychology, Institute of Psychology, Psychiatry and Neuroscience, King’s College London, London, UK; 3 Faculty of Health Sciences, University of Sydney, Sydney, Australia

**Keywords:** Williams syndrome, Anxiety disorders, Intellectual disability, Genetic syndromes, Meta-analysis, Systematic review

## Abstract

Individuals with specific genetic syndromes associated with intellectual disability (ID), such as Williams syndrome (WS), are at increased risk for developing anxiety disorders. A systematic literature review identified sixteen WS papers that could generate pooled prevalence estimates of anxiety disorders for WS. A meta-analysis compared these estimates with prevalence estimates for the heterogeneous ID population and the general population. Estimated rates of anxiety disorders in WS were high. WS individuals were four times more likely to experience anxiety than individuals with ID, and the risk was also heightened compared to the general population. The results provide further evidence of an unusual profile of high anxiety in WS.

**Electronic supplementary material:**

The online version of this article (doi:10.1007/s10803-016-2909-z) contains supplementary material, which is available to authorized users.

## Introduction

High rates of psychopathology and, in particular, elevated rates of anxiety disorders are characteristic of some genetic syndromes associated with intellectual disability (ID; Dekker et al. 2002; Dykens 2000). High levels of anxiety frequently result in disruption to and restriction of activities, impaired quality of life and the need for psychological services (Davies et al. 1998; Plissart et al. 1994). Associations between these genetic syndromes and anxiety disorders indicate a possible biological vulnerability, although the precise mechanisms are unknown (Jabbi et al. 2012). Thus, as well as being beneficial for diagnosis and intervention, knowledge about the phenomenology of anxiety in genetic syndromes could help identify the possible neural and genetic mechanisms involved. One syndrome with a reportedly high prevalence^1^ of anxiety disorders is Williams syndrome (WS), which affects approximately one in 7500 people (Stromme et al. 2002).

WS is caused by a sporadic microdeletion of 26–28 genes on chromosome 7q11.23 (Ewart et al. 1993; Jarvinen et al. 2013) and is associated with characteristic physical, cognitive, emotional, and behavioural traits (Morris 2010). The physical phenotype includes delayed development, distinguishing facial features, cardiovascular disease, hypercalcaemia, short stature, and supravalvular aortic stenosis (Morris and Mervis 2000). The majority of individuals have mild to moderate ID, with IQs typically ranging from 40 to 90 (Bellugi et al. 2000). The cognitive profile is uneven, with notable impairments in visuospatial processing skills but preserved expressive language and facial processing skills (Udwin and Yule 1991; Bellugi et al. 2000). WS is also associated with an unusual social phenotype, whereby individuals tend to have an extremely strong drive for social interaction (Jones et al. 2000). The emotional and behavioural difficulties associated with WS include anxiety, hyperactivity, impulsivity, distractibility, and disruptive behaviour (Einfeld et al. 1997, 2001; Gagliardi et al. 2011; Papaeliou et al. 2012; Udwin and Yule 1991).

Anxiety is one of the most dominant and persistent difficulties for individuals with WS, although there is considerable variability in reported prevalence estimates, with figures for any anxiety disorders ranging from 16.5 to 82.2 % (Stinton et al. 2010; Woodruff-Borden et al. 2010). This variability is likely the result of methodological differences between studies, in terms of the measures, diagnostic criteria, and samples (Dodd and Porter 2009; Green et al. 2012).

Despite discrepancies in estimates, the extent to which anxiety is elevated in WS relative to the general population is evident, with systematic reviews suggesting that global rates in the general population are around 7–11 % (Baxter et al. 2013; Somers et al. 2006). Moreover, although anxiety is a common feature in various genetic syndromes associated with ID (Emerson 2003), prevalence rates are typically higher in WS compared with a number of syndromes, including Prader–Willi syndrome, Down syndrome, and Fragile X syndrome (Pegoraro et al. 2014; Dykens et al. 2005). WS is also associated with higher rates of anxiety disorders compared with individuals with ID of mixed aetiology, with rates estimated at 3–22 % (Reardon et al. 2015). Thus, it seems that high levels of anxiety in WS may not be solely related to the presence of ID; instead these findings suggest a specific link between WS and a heightened vulnerability for the development of anxiety, which may be related to genes in the area of the deletion region (Dykens 2000). To our knowledge, there has been no systematic study of rates of anxiety disorders in WS compared with rates in other individuals with ID or in the general population. Therefore, we conducted a meta-analysis of the literature to estimate the quality-weighted pooled prevalence rates of anxiety disorders in WS and to compare the risk indices in WS with those for ID and the general population.

The aims of this review are to:

1. Amalgamate data from the existing literature and calculate the pooled prevalence estimates of anxiety disorders in WS and ID, taking into account the methodological quality of the studies involved.
2. Identify and evaluate the methods most frequently used for measuring anxiety prevalence in WS.
3. Compare pooled prevalence estimates in individuals with WS with estimates for individuals with ID of heterogeneous aetiology, and to compare each of these with general population estimates.

## Methodology

### Search Strategy and Selection Criteria

The review was designed in accordance with PRISMA guidelines (Moher et al. 2009). Five databases were selected for the systematic literature search; CINAHL (all years), Psychinfo (1967-April week 3 2015), Medline (1946-April week 3 2015), Embase (1974–2015 May 06), and Web of Science (all years). Appropriate search terms associated with WS were identified using medical subject headings (MeSH) definitions and genetics home reference terms. The terms, ‘william’ and ‘beuren’ were also included to widen search results. Search terms related to anxiety were derived from the Diagnostic and Statistical Manual of Mental Disorders, Fifth Edition (DSM-5) categories of anxiety (American Psychiatric Association [APA] 2013) and a literature review of anxiety in adults with ID (Hermans et al. 2011). The search was conducted using the search terms outlined in Table 1.

**Table 1:** Search terms used in electronic databases

	Search terms
Group A	beuren syndrome* OR elfin facies syndrome* OR elfin facies with hypercalcemia* OR hypercalcemia-supravalvar aortic stenosis* OR infantile hypercalcemia* OR supravalvar aortic stenosis syndrome* OR *WBS* OR williams beuren syndrome* OR *WMS* OR *WS* OR williams syndrome* OR chromosome 7q11.23 deletion syndrome* OR contiguous gene syndrome* OR williams contiguous gene syndrome* OR william* OR beuren*
Group B	anx* OR phobi* OR fear* OR panic disorder* OR worr* OR panic attack*

Group A and group B were combined with the term ‘AND’

### Study Selection

The multiple searches generated a total of 9201 references. The initial search phase utilised predefined inclusion and exclusion criteria to screen the titles and abstracts of generated results (see Table 2). In cases where eligibility was unclear, a second reviewer screened the information and agreement regarding inclusion was reached. The term ‘William’ generated numerous references that were not relevant to the syndrome under review, such as author’s names, models, and paradigms. Additionally, many studies did not explicitly reference anxiety and these studies were excluded. After the removal of duplicates, 80 relevant articles were retained.

**Table 2:** Phase one: Inclusion and exclusion criteria for screening titles and abstracts in preliminary search

Inclusion criteria	Exclusion criteria
Diagnosis of Williams syndrome Direct focus on anxiety Studies published in English Articles in peer reviewed journals	Non-human studies/mouse models Studies discussing the phenomenology of social functioning or emotional processing without a direct focus on anxiety Conference abstracts, conference papers, book chapters

**Fig. 1:**
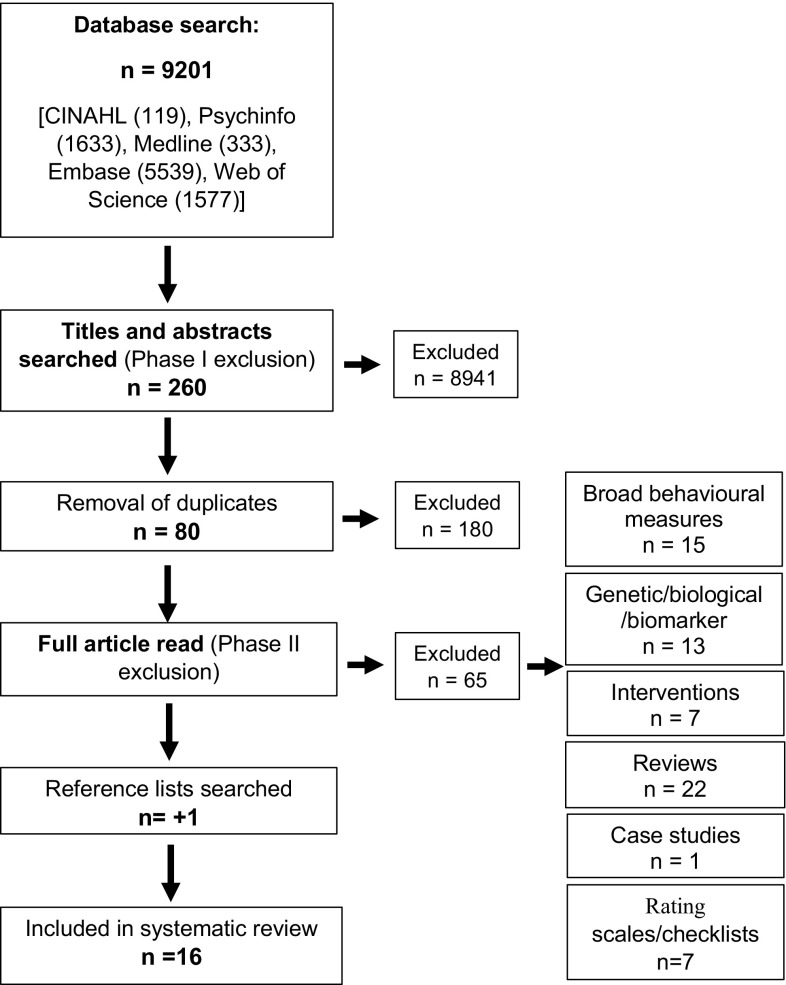
Search strategy

Following the initial screen, a second phase with more stringent inclusion criteria was implemented when reading the full text articles (Table 3). Only articles focusing on the prevalence rates of anxiety disorders were included (for full list of included and excluded studies, see Online Resource A). Through this process, 15 studies were identified as adhering to inclusion criteria. An additional manual scan of the articles’ reference lists identified one additional paper, resulting in a total of 16 papers. The complete search strategy is presented in Fig. 1.

**Table 3:** Phase two: inclusion and exclusion criteria used to assess full text articles

Inclusion criteria	Exclusion criteria
Studies with a psychiatric assessment/usage of DSM/ICD criteria Studies reporting anxiety disorder prevalence rates (including any anxiety disorder, specific phobias, generalised anxiety, separation anxiety, social anxiety, panic disorder, agoraphobia, obsessive–compulsive disorder and post-traumatic stress disorder)	Biological studies/genetic studies/biomarker studies Intervention studies Reviews Checklists/rating scales Studies using measures looking at a range of behaviours without anxiety as a focal point Case studies

### Quality Ratings

Methodological quality of the studies was rated using an adapted version of the criterion developed by Richards et al. (2015) (see Online Resource B for full details). Inter-rater reliability for the original version is good, (r(52) = 0.78, p < 0.001; Richards et al. 2015). Studies were rated on a scale from poor (score of ‘0’) to excellent (‘3’) on three core areas; sample identification, confirmation of syndrome diagnosis, and anxiety assessment used. The quality weightings were calculated by dividing the total quality score by the maximum score of nine. A second rater independently rated 37.5 % of the papers and inter-rater reliability was good (kappa = 0.68).

### Statistical Analysis

Estimated prevalence rates for anxiety disorders were extracted from the studies and pooled prevalence estimates were calculated using the statistical package MetaXL 2.0 (Barendregt and Doi 2011). This was used to generate both random-effects models and quality-effects models of anxiety disorder prevalence. The random-effects model, which accounts for variation between studies and estimates the mean of the distribution, was chosen over a fixed-effect model, which assumes studies share a common effect size (Borenstein et al. 2010). The quality weightings assigned to each study were also taken into consideration by calculating the quality-effects model (see Online Resource C for model summaries).

To account for studies involving overlapping cohorts of participants, only the data from the most methodologically robust study were retained. In cases where quality ratings did not differ, the study with the largest number of participants was included in the analysis.

Data from studies of WS were compared with pooled prevalence rates of anxiety disorders in ID of heterogeneous aetiology of both known and unknown origin; these latter figures were generated based on data reported in a recent systematic review by Reardon et al. (2015). Although the Reardon review (2015) primarily focuses on anxiety prevalence in children and adolescents (aged 5–20), it was, nevertheless, deemed the most appropriate comparison for the WS data, as 75 % of the WS studies included had a mean age of below 20 years. Although further matching of the cohorts (for age, cognitive level etc.) would have been preferable, without access to the raw data this option was unavailable. The seven papers in the systematic ID review by Reardon et al. (2015) used criteria from either the International Classification of Diseases (ICD-10; World Health Organisation [WHO] 1992) or the DSM-IV (APA 1994). The measures used included, the Development and Well-being Assessment (DAWBA; Goodman et al. 2000), the Diagnostic Interview Schedule for Children (DISC; Shaffer et al. 2000), and clinician interviews. For the purpose of the current review, the quality of the studies included in the Reardon et al. (2015) review were rated using the same methodology as for the WS papers. All of the ID papers were rated by two independent raters and inter-rater reliability was good (kappa = 0.79).

To compare the risks of having an anxiety disorder in WS and ID, relative risk statistics were calculated using the quality-effects pooled prevalence estimates and 95 % confidence intervals. WS and ID prevalence estimates were then compared using odds ratio statistics with pooled population estimates of any anxiety disorder from two reviews, a child and adolescent focused meta-analysis (Polanczyk et al. 2015), to match the WS and ID studies, and a systematic review and meta-regression inclusive of all age groups to reflect the general population (Baxter et al. 2013). Both reviews (Baxter et al. 2013; Polanczyk et al. 2015) utilised diagnostic procedures derived from the DSM or ICD (APA 1980, 1987, 1994; WHO 1978, 1992), and included community samples only. As there were no suitable reviews or pooled prevalence estimates for the individual categories of anxiety disorders, a nation-wide UK survey of approximately 8000 5–16 year olds (Green et al. 2005) was chosen to compare rates, using odds ratios with 95 % confidence intervals.

## Results

### Study Characteristics

Sixteen WS papers met the criteria for inclusion; eight of the studies were based in the US, four in Australia, two in Israel, one in the UK, and one in Brazil (Table 4). Publication dates of the papers identified ranged from 2003 to 2014. Five American studies (numbered 9, 10, 11, 12, 15 in Table 4) carried out assessments using the same cohort of participants, as did an additional four Australian studies (2–5). Seven (43.75 %) studies utilised samples of children under the age of 18, seven (43.75 %) used broad age ranges including both children and adults, and the remaining two papers (studies 1, 14) focused on adult samples only.

**Table 4:** Summary of included studies (sample characteristics and recruitment, anxiety measures used and quality scores)

Author	N	Gender	Mean age (range)	IQ mean (SD) or equivalent	Recruitment	Anxiety assessment	Quality score
1. Cherniske et al. (2004)	20	10 m, 10 f	38.8 years (30–51)	68 (SD unreported)	Genetics clinic at Yale New Haven Medical Center, website for the Yale Child Study Center Clinic for Genetic Forms of Developmental Disorders, WSA, clinical colleagues, USA	ADIS, open-ended interviews K-SADS	0.78
2. Dodd and Porter (2009)	50	24 m, 26 f	18.53 years (6–50)	Mental age: 6.25, range: 2.16–10.58	WSA, Australia	K-SADS-PL	0.78
3. Dodd and Porter (2011a)	16	9 m, 7 f	21 years (13–34.9)	Mental age: 8.08 (SD: 1.04)	WSA, Australia	K-SADS-PL	0.89
4. Dodd and Porter (2011b)	16	9 m, 7 f	21.04 years (13–34)	Mental age: 8.09 (SD: 1.04)	WSA, Australia	K-SADS-PL	0.67
5. Dodd et al. (2009)	15	11 m, 6 f	19.6 years (12–28)	Mental age: 8.20, range: 7–10	WSA, Australia	K-SADS-PL	0.67
6. Dykens (2003)	51	23 m, 28 f	15.91 years (5–49)	62.0 (SD: 15.44)	Far west chapter of the National WSA, referrals from university-based geneticists, 1998 WSA Biennial Meeting, USA	DICA–R	0.89
7. Green et al. (2012)	38	16 m, 22 f	13.1 years (16–23)	63.3 (SD: 11.4)	Hospital clinical genetics department, Israel	K-SADS	0.78
8. Kennedy et al. (2006)	21	7 m, 14 f	16 years (7–28)	Unreported	Williams Syndrome Clinic of Women and Children’s Hospital of Buffalo, USA	ADIS	0.78
9. Leyfer et al. (2012)	192	87 m, 105 f	7.28 years (5.01–10.94)	Mean: 75.59 (SD: 15.32)	Part of a longitudinal study, USA	ADIS-P^a^	0.67
10. Leyfer et al. (2009)	132	63 m, 69 f	8.5 years (4–16.9)	DAS GCA Mean: 60.2 (SD: 13.6)	Ongoing study of language and cognitive development, USA	ADIS-P	0.78
11. Leyfer et al. (2006)	119	54 m, 65 f	9.1 years (4.01–16.9)	DAS GCA Mean: 59.5 (SD: 13.7)	Ongoing study of language and cognitive development, University of Wisconsin-Milwaukee, USA	ADIS-P	0.89
12. Mervis et al. (2012)	214	107 m, 107 f	8.19 years (4.07–12.96)	Unreported	Part of a larger study, USA	ADIS-P	0.67
13. Pegoraro et al. (2014)	10	7 m, 3 f	11.7 years (6–16)	Mean: 58.9 (SD: 5.9)	Outpatient clinics, specialist institution, Brazil	Assessment by trained psychiatrist (DSM-IV criteria)	0.56
14. Stinton et al. (2010)	92	50 f, 42 m	32 years (19–55)	Mean: 56.6 (SD: 7.2)	WSF, UK	PAS-ADD	0.67
15. Woodruff-Borden et al. (2010)	45	21 m, 24 f	6.67 years (4–13.42)	Median IQ Without anxiety: 79.1, with anxiety: 78.2	Ongoing longitudinal study of cognitive and language development, USA	ADIS-P	0.67
16. Zarchi et al. (2014)	24	10 m, 14 f	16.8 years (not reported)	Mean: 66.59 (SD: 9.55)	Behavioural Neurogenetics Center, Israel	KSADS-PL	0.67

*DAS GCA* differential ability scales general conceptual ability, *WSA* Williams Syndrome Association, *WSF* Williams Syndrome Foundation, *ADIS-(P)* anxiety disorder interview schedule–(parent version), *K-SADS-(PL)* Kiddie schedule of affective disorders (present and lifetime version), *DICA-R* diagnostic interview schedule for children, *DSM-IV* diagnostic and statistical manual of mental disorders 4th edition, *PAS-ADD* psychiatric assessment schedule for adults with developmental-disabilities

^a^Administered to subset of sample *n* = 109

A total of 1055 participants was included in the WS meta-analysis; however, after accounting for individuals included in the overlapping cohorts, numbers reduce to 391 participants. The mean sample size of all the included studies was *n* = 66 (SD 65.3; range 10–214), with an average male to female ratio of 32:34. The average age of the participants was 16.5 years (SD 8.9, range 4–55). Seven studies reported on the IQ level of participants; the mean was 64.43, SD = 6.34 (range 56.6–75.6).

### Quality Ratings

All WS studies failed to obtain the highest quality rating score of nine, however two (studies 3, 11) scored eight. The majority of papers (15, 93.8 %) obtained a score of three for syndrome confirmation but no studies achieved this score for sample identification. This was due to studies recruiting from single or multiple research sites, and not from a random or total population sample, as was required for the maximum score. However, given the rarity of the syndrome, this method of sampling is not a feasible option. Quality scores for anxiety assessments were variable, with only five (31.3 %) studies (1, 3, 6, 8, 11) attaining the highest rating. This was achieved through reaching consensus using multiple measures, including at least one diagnostic assessment. All of the included studies were rated as being of ‘adequate’ or ‘good’ quality.

### Anxiety Measures Used

Four standardised psychiatric assessments were used in the reviewed WS papers; two versions of the Kiddie Schedule of Affective Disorders (KSADS; Kaufman et al. 1997), the Anxiety Disorder Interview Schedule (ADIS; Silverman and Albano 1996), the Diagnostic Interview Schedule for Children–Parent Version (DICA–R; Reich et al. 1991) and the Psychiatric Assessment Schedule for Adults with Developmental Disabilities (PAS-ADD; Moss et al. 1996). The KSADS and ADIS were the most frequently used psychiatric assessments, each used in seven studies. The most commonly used version of the KSADS, the present and lifetime version, has good test–retest reliability (present diagnoses, kappa = 0.74; lifetime diagnoses, kappa = 0.60) and strong inter-rater agreement (mean agreement = 98 %, range = 93–100 %; Kaufman et al. 1997). The ADIS also has strong psychometric properties; test–retest reliability is excellent (ICC = 0.81–0.96) and the reliability of anxiety disorder diagnoses range from good to excellent (kappa = 0.65–0.88; Silverman et al. 2001). Of the remaining measures, the PAS-ADD, the only ID specific measure, is less robust, with particularly the anxiety disorder section being rated as having low validity (Moss et al. 1997). The DICA-R is based on an earlier version of the DSM and has been shown to have poor test–retest reliability for some anxiety disorders (kappa = 0.38–0.46; Boyle et al. 1993) and poor concordance with clinician judgements for specific phobias, post-traumatic stress disorder and obsessive–compulsive disorder (Ezpeleta et al. 1997).

The measures provide both informant and self-report elements, and there was variation in the versions used between studies. Eleven of the studies obtained data by interviewing primary caregivers only; the remaining five (studies 1, 7, 8, 14, 16), used a combination of both informant and respondent interviews.

### Prevalence Estimates and Profiles of Anxiety Disorders

The majority of papers (14, 87.5 %) used anxiety assessments that adhered to DSM-IV criteria (APA 1994). As a result, this review categorised disorders according to this classification, rather than the more recent version (DSM-5; APA 2013). The random-effects and quality-effects pooled prevalence estimates of anxiety disorders are reported in Table 5 (for all forest plots see Online Resource D and E). The data indicate that 48 % (95 % CI 26.0–70.0) of individuals included in the review experienced at least one anxiety disorder. The most prevalent disorder diagnosed was specific phobias (identified in nine studies; quality-effects 39 %), and there were commonalities across studies regarding the main phobias reported (Table 6). Among the top three phobias reported in each study, the most frequent phobia was noise (*n* = 6); followed by, blood, injury or injection (*n* = 4); thunderstorms/lightning (*n* = 3); animals (*n* = 3); and the category ‘other’ (*n* = 3). Generalised anxiety disorder (GAD) was also relatively common, with estimated rates of 10 % (95 % CI 4.0–19.0). The remaining anxiety disorders were less common, with the lowest estimate for social anxiety disorder (quality-effects 1 %).

**Table 5:** Summary of quality ratings for each study (mean quality weightings; percentages of studies obtaining top scores for each criterion; percentages of studies with quality ratings of ‘poor’, ‘adequate’, ‘good’, and random-effects/quality effects models with 95 % confidence intervals)

	Included studies (N)	Total Ppts (N)	Mean QW	% obtained score of 3 for sample (N)	% obtained score of 3 for syndrome (N)	% obtained score of 3 for anxiety assessment (N)	% ‘poor’ QW (N)	% ‘adequate’ QW (N)	% ‘good’ QW (N)	Random-effects pooled prev. (CI)	Quality-effects pooled prev. (CI)
Any anxiety disorder	7^b^	333	0.73	0.0 (0)	100.0 (7)	28.6 (2)	0.0 (0)	42.9 (3)	57.1 (4)	48.0 (3.0–67.0)	48.0 (26.0–70.0)
Specific phobias	9^b^	391	0.77	0.0 (0)	88.9 (8)	55.6 (5)	0.0 (0)	33.3 (3)	66.7 (6)	40.0 (27.0–54.0)	39.0 (24.0–55.0)
GAD	7^b^	361	0.81	0.0 (0)	100.0 (7)	57.1 (4)	0.0 (0)	28.6 (2)	71.4 (5)	11.0 (5.0–19.0)	10.0 (4.0–19.0)
Separation AD	6^b^	303	0.82	0.0 (0)	100.0 (6)	50.0 (3)	0.0 (0)	16.7 (1)	83.3 (5)	7.0 (2.0–15.0)	7·0 (1.0–15.0)
Social AD	6^b^	344	0.78	0.0 (0)	100.0 (6)	33.3 (2)	0.0 (0)	33.3 (2)	66.7 (4)	1.0 (0.0–3.0)	1·0 (0.0–3.0)
PD	6^b^	340	0.80	0.0 (0)	83.3 (5)	50.0 (3)	0.0 (0)	16.7 (1)	83.3 (5)	2.0 (1.0–4.0)	2.0 (1.0–4.0)
PTSD	4^b^	202	0.78	0.0 (0)	100.0 (4)	50.0 (2)	0.0 (0)	25.0 (1)	75.0 (3)	2.0 (0.0–4.0)	2.0 (0.0–4.0)
Ag^a^	3	163	0.74	0.0 (0)	100.0 (3)	33.3 (1)	0.0 (0)	33.3 (1)	66.7 (2)	2.0 (0.0–5.0)	2.0 (1.0–6.0)
OCD	7^b^	289	0.83	0.0 (0)	85.7 (6)	71.4 (5)	0.0 (0)	14.3 (1)	85.7 (6)	4.0 (2.0–6.0)	4.0 (2.0–7.0)

Quality weightings derived from categories outlined by Richards et al. (2015); ‘poor’ (0.33–0.55), ‘adequate’ (0.56–0.77) and ‘good’ (0.78–1.0)

*GAD* generalised anxiety disorder, *AD* anxiety disorder, *PD* panic disorder, *PTSD* post-traumatic stress disorder, *Ag* agoraphobia, *OCD* obsessive compulsive disorder, *QW* quality weightings

^a^May not represent true figures. Agoraphobia was sometimes grouped with specific phobias (n = 1) and panic disorders (n = 1)

^b^Studies with overlapping cohorts removed, study with the highest quality rating retained

**Table 6:** Prevalence rates of anxiety disorders in WS, as reported in the 16 included studies

Author	Reported anxiety rates								
	At least one anxiety disorder	Specific phobias	GAD	Separation AD	Social AD	PD	PTSD	Ag^a^	OCD
1. Cherniske et al. (2004)	65 % moderate-severe 15 % mild	50 %	2nd most common diagnosis^b^	–	–	5 %	–	–	5 %
2. Dodd and Porter (2009)	38 %	30 %^c^ (natural environment: 18 %, noise: 12 %, blood-injury: 6 %)^d^	10 %	0 %	0 %	0 %	–	2 %	4 %
3. Dodd and Porter (2011a)	43.75 %	31.25 %	6.25 %	–	–	–	–	–	6.25 %
4. Dodd and Porter (2011b)	43.75 %	37.5 %	6.25 %	–	–	–	–	–	–
5. Dodd et al. (2009)	–	40 %	6.7 %	–	–	–	–	–	–
6. Dykens (2003)	–	35 % (natural environment: 94 %, other: 44 %, animals: 22 %)^d^	18 %	4 %	–	–	–	–	2 %
7. Green et al. (2012)	65.8 %	44.7 % (noises: 36.8 %, blood, injection: 15.9 %, other: 10.5 %)^d^	15.8 %	26.3 %	0 %	0 %	0 %	–	7.9 %
8. Kennedy et al. (2006)	48 %	43 % (animals, thunderstorms/lightning, loud noises)^d^	24 %	5 %	0 %	5 %	5 %	5 %	0 %
9. Leyfer et al. (2012)	69.7 %	53.2 %	–	–	0 %	–	–	–	–
10. Leyfer et al. (2009)	62.1 %	56.1 %	7.6 %	6.1 %	2.3 %	–	1.5 %	–	1.5 %
11. Leyfer et al. (2006)	–	53.8 % (loud noises: 27.7 %), blood tests/shots: 15.9 %, doctor/dentist: 8.4 %)^d^	11.8 %	6.7 %	1.7 %	0.8 %^c^	0.8 %	–	2.5 %
12. Mervis et al. (2012)	–	–	–	4.2 %	–	–	–	–	–
13. Pegoraro et al. (2014)	60 %	60 %	–	–	–	–	–	–	–
14. Stinton et al. (2010)	16.5 % (*n* = 75)	12.1 % (fear of storms: 54.5 %, hospitals: 18 %)^d^	1.1 %	–	2.2 %	3.3 %	–	4.4 %	–
15. Woodruff-Borden et al. (2010)^e^	82.2 %	51.1 % (loud noises: 60 %, other: 42.2 %, blood-injury: 40 %)^d^	15.6 %	11.1 %	6.7 %	0 %	0 %	–	2.2 %
16. Zarchi et al. (2014)	50 %	45.8 %	8.3 %	12.5 %	0 %	–	4.2 %	–	4.2 %

*GAD* generalised anxiety disorder, *AD* anxiety disorder, *PD* panic disorder, *PTSD* post-traumatic stress disorder, *Ag* agoraphobia, *OCD* obsessive compulsive disorder

^a^When reported independently

^b^Prevalence unreported

^c^includes agoraphobia

^d^Top three phobias reported

^e^Taken at time-point 1

Generated prevalence estimates for anxiety disorders in WS were compared with ID population rates using relative risk analyses. The results indicate that individuals with WS were significantly more likely to have an anxiety disorder [risk ratio (RR) 4.00 (95 % CI 2.27–7.06); p < 0.0001], and in particular, to have a specific phobia [RR 5.57 (95 % CI 2.62–11.86); p < 0.0001] or GAD [RR 10.00 (95 % CI 1.30–76.67); p < 0.05], than individuals with heterogeneous ID (for full table of results, see Online Resource F).

Odds ratio statistics were used to compare rates of anxiety disorders in WS and ID with general population rates. The odds of an anxiety disorder was significantly more likely in WS compared with child and adolescent population rates [Odds ratio (OR) 13.28, 95 % CI 5.47–32.22; p < 0.05] and all ages general population rates [OR 11.72, 95 % CI 5.01–27.41; p < 0.05]. There were no significant differences in the risk of having anxiety in the ID population compared to child/adolescent and general population rates.

Odds ratios statistics with 95 % confidence intervals were generated to compare WS and ID quality-effects prevalence estimates of individual anxiety disorders with UK national child population estimates. Using these estimates, having an anxiety disorder was significantly more likely in WS (OR 27.05, 95 % CI 8.44–86.74; p < 0.05), as well as in ID (OR 4.00, 95 % CI 1.14–13.98; p < 0.05), compared with population estimates, although the odds were much higher for individuals with WS. Moreover, the odds of having a specific phobia (OR 79.28, 95 % CI 8.47–742.13; p < 0.05) or GAD (OR 13.78, 95 % CI 1.39–136.75; p < 0.05) were significantly more likely in WS compared with the UK child population, although no differences were found for ID (see Online Resource F for further details).

## Discussion

This is the first meta-analytical review to generate direct comparisons between rates of anxiety disorders in individuals with a specific genetic syndrome (WS) and those with heterogeneous ID, and population estimates. Random-effects and quality-effects models were generated for WS and ID using the available WS literature and a pre-existing ID systematic review respectively, and statistical analysis of risk was used to compare estimates. The rate of anxiety disorder in individuals with WS was calculated at approximately 48 %, a significantly higher figure than the 12 % estimated in the child ID population, and the variable yet lower estimates reported in the general population (Baxter et al. 2013; Somers et al. 2006). Unexpectedly, the likelihood of developing an anxiety disorder in ID did not seem to be elevated compared to the general population, contrary to previous findings (Deb et al. 2001). However, as this review indicates, results are heavily dependent on the choice of comparison estimates. For example, there were discrepancies between the odds calculated with the two global prevalence review papers (Baxter et al. 2013; Polanczyk et al. 2015) compared with the UK national survey (Green et al. 2005). The reported rates of anxiety disorders in the study by Green et al. (2005) are low and thus may have inflated the differences in risk between the child population and ID group. These relatively low figures may be due to the fact that the Green et al. (2005) study relied entirely on parental reports of diagnosed disorders, which could potentially exclude a high proportion of individuals with undiagnosed anxiety. The significant variability in reported general population estimates is also a limitation of much anxiety research, and is often attributed to the representativeness and frame of the sample and the choice of diagnostic instrument (Polanczyk et al. 2015). As a result, comparative analyses in this context should be interpreted cautiously. Further investigation is essential to decipher the relationship between ID and anxiety, as it remains unclear whether or not the presence of ID increases the likelihood of developing an anxiety disorder.

In contrast, it is evident that there is a strong relationship between WS and the presence of anxiety disorders, and this association is mainly attributable to two categories of anxiety disorder: specific phobias and GAD. Specific phobias were the most prevalent anxiety disorder reported in the WS samples (estimated at 39 %). Although these rates were slightly elevated in the ID group (7 %) compared with UK child population estimates (0.8 %), they were much lower than risk estimates in WS. The content of the phobias also appeared to be distinctive in WS, with reported phobias often relating to noise stimuli and blood, injury and injections, whereas the most commonly reported phobias in other studies of individuals with ID have included fears of ghosts and animals (Dykens 2003; Green et al. 2012). Certain phobias experienced by people with WS may be related to some of the phenotypic characteristics of the disorder, for example, a heightened sensitivity to auditory stimuli (hyperacusis) and frequent hospitalisations/health problems may lead to fears of loud noises and of blood/injury (Dykens 2003). However, it is possible that reported rates of specific phobias are misleading, particularly in relation to the prevalence of noise phobias. For those with hyperacusis (which is estimated to affect approximately 95 % of the WS population; Klein et al. 1990), loud noises are very aversive and can cause pain. Since irrationality is a core feature of the definition of a phobia (APA 2013), fear of noise may not be an irrational response, and if such fears are incorrectly classified as phobias, this could result in an overestimation in specific phobia prevalence rates. This issue is widely debated, and although it seems unlikely that noise phobias can fully account for the high rates of reported phobias, future research should consider comparing the prevalence of phobias in WS with and without the inclusion of noise. Hyperacusis is also reported to decrease with age (Gothelf et al. 2006), therefore investigating whether noise phobias are present at similar rates in the adult WS population may shed further light on whether this constitutes a true phobia in WS.

The high rate of GAD among WS participants was also notable. Rates of GAD in ID samples were low (1 %) but the risk of GAD increased ten-fold for individuals with WS. The high rates of phobias and GAD suggest that specific types of anxiety problems may be strongly associated with the genetic aetiology of WS, rather than with the presence of ID per se. Preliminary evidence for this vulnerability stems from studies examining neurological and structural brain differences in WS. Structural deficits in white matter pathways have been implicated in the heightened amygdala activation observed for threatening stimuli in individuals with WS (Avery et al. 2011; Meyer-Lindenberg et al. 2005; Munoz et al. 2010; Thornton-Wells et al. 2011). Such abnormalities may result in increased prevalence of anxiety in WS, although further research to investigate the underlying mechanisms is warranted. In terms of specific anxiety disorders, the GFT21 gene has been linked with dorsolateral prefrontal cortex activation and anxiety proneness in typically developing individuals (Jabbi et al. 2015). This gene is hemizygously deleted in WS and is suggested to account for the hyper-sociability and lower rates of social anxiety that are characteristic of WS (Schubert 2009; Sakurai et al. 2011). Additional research to investigate this association further is essential, as is exploration of the genetic influences of other anxiety disorders in WS, particularly specific phobias and GAD. This will enhance understanding of the roles and contributions of genetic and neural mechanisms in the development of anxiety disorders in WS, as well as in the general population.

Although many of the studies included in our review utilised similar psychometrically robust measures that correspond to the same classification system (i.e. DSM-IV), comparability of prevalence rates between the papers should be interpreted carefully. Whilst the DSM tends to increase the number of diagnostic categories included in each revision, the ICD has remained more stable over recent editions (Cooper et al. 2003; Tyrer 2014). This may account for the lower anxiety prevalence rates reported by Stinton et al. (2010), and may have contributed to a lower pooled prevalence of anxiety for the ID studies. The choice of classification system has been shown to produce differing estimates (Slade and Andrews 2001), demonstrating the importance of considering the measures and classifications used when interpreting results. Future studies introducing DSM-5 categorisation should also bear the likely discrepancies with previous criteria in mind.

### Study Limitations

Out of the included studies, 37.5 % recruited participants from clinical settings. This may have inflated the prevalence estimates reported. In addition, the review was unable to match participants in the ID vs. WS comparative analyses, and so it is difficult to evaluate whether group differences may account for the differing anxiety rates reported. Einfeld and Tonge (1996) found a positive correlation between IQ and anxiety in individuals with ID, and since WS is mostly associated with a milder degree of ID (Bellugi et al. 2000), higher anxiety rates may be related to higher IQ levels. Additionally, WS is associated with an increased verbal ability, relative to other genetic syndromes and other forms of ID (Bellugi et al. 1990; Brock 2007; Pegoraro et al. 2014). Thus, individuals with WS may be more able than others to express their internalising thoughts and feelings, which may lead to increased diagnosis (Ng et al. 2014). Nevertheless, the findings are consistent with other comparative studies indicating that anxiety disorders in WS are significantly more common than in heterogeneous ID groups.

The large confidence intervals generated in the analysis also reflect the current lack of methodologically robust studies in this area, which limits the ability to generate more precise prevalence estimates. The development of more stringent quality criteria is needed to enhance the reliability of studies and to improve knowledge about the relative risks and profiles of anxiety in individuals with ID and genetic syndromes.

### Clinical and Research Implications

This review has identified several key limitations with the existing WS literature. The identification of specific anxiety disorder profiles in WS suggests that the use of the category of ‘any anxiety disorder’ in the literature may be misleading. Such descriptions lead to the assumption that there is a fairly even distribution of anxiety disorders in WS, but this is clearly not supported by the present analysis. Research reporting the individual rates of each anxiety disorder for individuals with WS and other genetic syndromes is needed to identify and target syndrome-specific difficulties, as well as to identify between group similarities and differences.

A broader issue with existing research is the use of diagnostic classification criteria developed for the general population, which may not be appropriate or sensitive enough to diagnose anxiety disorders in ID (Cooper et al. 2003; Szymanski 1994). Current criteria often require self-reporting of internalising symptoms and this can be challenging for many individuals (Deb et al. 2001). Consequently, reported prevalence estimates may be a misrepresentation of true rates of anxiety disorders in these groups. The clinical presentation of anxiety in ID may also differ from the typically developing population and hence, existing classification systems may be missing important symptoms (Khreim and Mikkelsen 1997). The use of the category “Other”, which was one of the most common categories of specific phobia reported in the review, should be avoided as this may conceal information that is vital to our understanding of anxiety in WS.

Additionally, it is difficult to draw definitive conclusions regarding the prevalence rates of anxiety in the WS population due to differences in sample selection across studies and also the underrepresentation of adult participants. Since GAD has been reported to increase with age in WS (Dodd and Porter 2009), existing data provide little information about anxiety trajectories across the lifespan.

In conclusion, this review confirms the heightened risks to individuals with WS of developing an anxiety disorder and indicates this risk cannot be accounted for by the presence of ID. The review also highlights the importance of investigating specific profiles of anxiety, as well as overall rates, in syndrome groups. Further research should focus on the genetic mechanisms underpinning anxiety, investigating developmental trajectories of anxiety and hyperacusis, and the creation of targeted interventions for syndrome related forms of anxiety. For individuals with WS, further examination of the phenomenology of anxiety and the effectiveness of interventions targeted towards specific phobias and GAD would seem to be particularly beneficial.

## Electronic supplementary material

Below is the link to the electronic supplementary material.

Supplementary material 1 (PDF 599 KB)

## Footnotes

1 The term prevalence is used to describe the “number of cases of… [a] condition, present at a particular time, in relation to the size of the population from which it is drawn” (Timmreck 2002, p. 151).
