# Supplementary material for: Anxiety Disorders in Williams Syndrome Contrasted with Intellectual Disability and the General Population: A Systematic Review and Meta-Analysis
Source: J Autism Dev Disord. 2016 Sep 30;47(12):3765–77. doi: 10.1007/s10803-016-2909-z (PMC5676825; doi:10.1007/s10803-016-2909-z)
Supplement: Supplementary file 1 — Supplementary material 1 (PDF 599 KB) [file 10803_2016_2909_MOESM1_ESM.pdf]

Anxiety Disorders in Williams Syndrome Contrasted with Intellectual Disability and the General Population: A Systematic Review and Meta-Analysis

Royston, R., Howlin, P., Waite, J. & Oliver, C.

Rachel Royston - BSc, University of Birmingham

Patricia Howlin - PhD, Kings College London & University of Sydney

Jane Waite - PhD, University of Birmingham

Chris Oliver - PhD, University of Birmingham

Corresponding Author: Rachel Royston
The Cerebra Centre for Neurodevelopmental Disorders,
School of Psychology, University of Birmingham,
Edgbaston, Birmingham, B15 2TT
Email: rxr180@bham.ac.uk
Telephone: 0121 414 2855

Online Resource A

Complete list of papers based on the full text search for the systematic review

Included Articles

1. Cherniske EM, Carpenter TO, Klaiman C, et al. Multisystem study of 20 older adults with Williams syndrome. *American Journal of Medical Genetics Part A*. 2004; **131A** (3): 255-64.
2. Dodd HF, Porter MA. Psychopathology in Williams syndrome: The effect of individual differences across the life span. *Journal of Mental Health Research in Intellectual Disabilities*. 2009;**2**(2):89-109.
3. Dodd HF, Porter MA. There's that scary picture : Attention bias to threatening scenes in Williams syndrome. *Neuropsychologia*. 2011;**49, 2**:247-53.
4. Dodd HF, Porter MA. Interpretation of Ambiguous Situations : Evidence for a Dissociation Between Social and Physical Threat in Williams Syndrome. *Journal of Autism and Developmental Disorders*. 2011;**41, 3**:266-74.
5. Dodd HF, Schniering CA, Porter MA. Beyond Behaviour: Is Social Anxiety Low in Williams Syndrome? *Journal of Autism and Developmental Disorders*. 2009; **39**(12):1673-81.
6. Dykens EM. Anxiety, fears, and phobias in persons with Williams syndrome. *Developmental Neuropsychology*. 2003;**23**(1-2):291-316.
7. Green T, Avda S, Dotan I, et al. Phenotypic psychiatric characterization of children with Williams syndrome and response of those with ADHD to methylphenidate treatment. *Am J Med Genet, Part B-Neuropsychiatric Genetics*. 2011;**159B**(1):13-20.
8. Kennedy JC, Kaye DL, Sadler LS. Psychiatric Diagnoses in Patients with Williams Syndrome and Their Families. *Jefferson Journal of Psychiatry*. 2006;**20**(1):22-31.
9. Leyfer O, John AE, Woodruff-Borden J, Mervis CB. Factor Structure of the Children’s Behavior Questionnaire in Children with Williams Syndrome. *Journal of Autism and Developmental Disorders*. 2012;**42, 11**: 2346-53.
10. Leyfer O, Woodruff-Borden J, Mervis CB. Anxiety disorders in children with Williams syndrome, their mothers, and their siblings : Implications for the etiology of anxiety disorders. *Journal of Neurodevelopmental Disorders*. 2009;**1, 1**: 4-14.
11. Leyfer OT, Woodruff-Borden J, Klein-Tasman BP, Fricke JS, Mervis CB. Prevalence of psychiatric disorders in 4 to 16-year-olds with Williams syndrome. *American Journal of Medical Genetics Part B-Neuropsychiatric Genetics*. 2006; **141B**(6):615-22.
12. Mervis CB, Dida J, Lam E, et al. Duplication of GTF2I results in separation anxiety in mice and humans. *American Journal of Human Genetics*. 2012; **90**(6):1064-70.
13. Pegoraro LFL, Steiner CE, Celeri EHRV, Banzato CEM, Dalgalarrondo P. Cognitive and behavioral heterogeneity in genetic syndromes. *Jornal de Pediatria*. 2014;**90**(2):155-60.
14. Stinton C, Elison S, Howlin P. Mental Health Problems in Adults With Williams Syndrome. *Ajidd-American Journal on Intellectual and Developmental Disabilities*. 2010;**115**(1):3-18.
15. Woodruff-Borden J, Kistler DJ, Henderson DR, Crawford NA, Mervis CB. Longitudinal Course of Anxiety in Children and Adolescents With Williams Syndrome. *Am J Med Genet, Part C-Seminars in Medical Genetics*. 2010;**154C**(2):277-90.
16. Zarchi O, Diamond A, Weinberger R, et al. A comparative study of the neuropsychiatric and neurocognitive phenotype in two microdeletion syndromes:

Velocardiofacial (22q11.2 deletion) and Williams (7q11.23 deletion) syndromes. *European Psychiatry*. 2014;**29**(4):203-10.

Excluded Studies

1. Annaz D, Hill CM, Ashworth A, Holley S, Karmiloff-Smith A. Characterisation of sleep problems in children with Williams syndrome. *Research in Developmental Disabilities*. 2011;**32**(1):164-9.
2. Ashworth A, Hill CM, Karmiloff-Smith A, Dimitriou D. Cross syndrome comparison of sleep problems in children with Down syndrome and Williams syndrome. *Research in Developmental Disabilities*. 2013;**34**(5):1572-80.
3. Avery SN, Thornton-Wells TA, Anderson AW, Blackford JU. White matter integrity deficits in prefrontal-amygdala pathways in Williams syndrome. *Neuroimage*. 2012;**59**(2):887-94.
4. Binelli C, Subira S, Batalla A, et al. Common and distinct neural correlates of facial emotion processing in social anxiety disorder and Williams syndrome: A systematic review and voxel-based meta-analysis of functional resonance imaging studies. *Neuropsychologia*. 2014;**64**:205-17.
5. Blomberg S, Rosander M, Andersson G. Fears, hyperacusis and musicality in Williams syndrome. *Research in Developmental Disabilities*. 2006;**27, 6**:668-80.
6. Bodizs R, Gombos F, Gervan P, Szocs K, Rethelyi JM, Kovacs I. Aging and sleep in Williams syndrome: Accelerated sleep deterioration and decelerated slow wave sleep decrement. *Research in Developmental Disabilities*. 2014;**35**(12):3226-35.
7. Cohen JD, Mock JR, Nichols T, et al. Morphometry of human insular cortex and insular volume reduction in Williams syndrome. *Journal of Psychiatric Research*. 2010;**44**(2):81-9.
8. Crespi BJ, Hurd PL. Cognitive-behavioral phenotypes of Williams syndrome are associated with genetic variation in the GTF2I gene, in a healthy population. *Bmc Neuroscience*. 2014;**15**.
9. Dai L, Carter CS, Ying J, Bellugi U, Pournajafi-Nazarloo H, Korenberg JR. Oxytocin and Vasopressin Are Dysregulated in Williams Syndrome, a Genetic Disorder Affecting Social Behavior. *Plos One*. 2012;**7**(6).
10. Davies M, Udwin O, Howlin P. Adults with Williams syndrome - Preliminary study of social, emotional and behavioural difficulties. *Br J Psychiatry*. 1998;**172**:273-6.
11. Donnai D, Karmiloff-Smith A. Williams syndrome: From genotype through to the cognitive phenotype. *American Journal of Medical Genetics*. 2000;**97**(2):164-71.
12. Dykens EM. Intervention issues in persons with Williams Syndrome. *Mental Health Aspects of Developmental Disabilities*. 2001;**4**(4):130-7.
13. Dykens EM, Rosner BA, Ly T, Sagun J. Music and anxiety in Williams syndrome: A harmonious or discordant relationship? *Am J Ment Retard*. 2005;**110**(5):346-58+413.
14. Einfeld SL, Tonge BJ, Florio T. Behavioral and emotional disturbance in individuals with Williams syndrome. *Am J Ment Retard*. 1997;**102**(1):45-53.
15. Einfeld SL, Tonge BJ, Rees VW. Longitudinal course of behavioral and emotional problems in Williams syndrome. *Am J Ment Retard*. 2001;**106**(1):73-81.
16. Eisenberg DP, Jabbi M, Berman KF. Bridging the gene-behavior divide through neuroimaging deletion syndromes: Velocardiofacial (22q11.2 Deletion) and Williams (7q11.23 Deletion) syndromes. *Neuroimage*. 2010;**53**(3):857-69.

17. Freeman K, Williams TI, Ferran E, Brown J. Williams Syndrome: The extent of agreement between parent and self report of psychological. *European Journal of Psychiatry*. 2010;**24**(3):167-75.
18. Gagliardi C, Martelli S, Tavano A, Borgatti R. Behavioural features of Italian infants and young adults with Williams-Beuren syndrome. *J Intellect Disabil Res*. 2011;**55, 2**:121-31.
19. Gosch A, Pankau R. Social-emotional and behavioral adjustment in children with Williams- Beuren syndrome. *American Journal of Medical Genetics*. 1994;**53**(4):335-9.
20. Graham JM, Rosner B, Dykens E, Visootsak J. Behavioral features of CHARGE syndrome (Hall-Hittner syndrome) comparison with Down syndrome, Prader-Willi syndrome, and Williams syndrome. *American Journal of Medical Genetics Part A*. 2005;**133A**(3):240-7.
21. Graham JM, Superneau D, Rogers RC, Corning K, Schwartz CE, Dykens EM. Clinical and behavioral characteristics in FG syndrome. *American Journal of Medical Genetics*. 1999;**85**(5):470-5.
22. Haas BW, Hoeft F, Searcy YM, Mills D, Bellugi U, Reiss A. Individual differences in social behavior predict amygdala response to fearful facial expressions in Williams syndrome. *Neuropsychologia*. 2010;**48**(5):1283-8.
23. Haas BW, Mills D, Yam A, Hoeft F, Bellugi U, Reiss A. Genetic Influences on Sociability: Heightened Amygdala Reactivity and Event-Related Responses to Positive Social Stimuli in Williams Syndrome. *Journal of Neuroscience*. 2009;**29**(4):1132-9.
24. Haas BW, Reiss AL. Social brain development in Williams syndrome: The current status and directions for future research. *Frontiers in psychology*. 2012;**3**:186.
25. Jabbi M, Kippenhan JS, Kohn P, et al. The Williams syndrome chromosome 7q11.23 hemideletion confers hypersocial, anxious personality coupled with altered insula structure and function. *Proceedings of the National Academy of Sciences of the United States of America*. 2012;**109**(14):E860-E6.
26. Jarvinen A, Korenberg JR, Bellugi U. The social phenotype of Williams syndrome. *Curr Opin Neurobiol*. 2013;**23**(3):414-22.
27. Kippenhan JS, Olsen RK, Mervis CB, et al. Genetic contributions to human gyrification: Sulcal morphometry in Williams syndrome. *Journal of Neuroscience*. 2005;**25**(34):7840-6.
28. Kirk HE, Hocking DR, Riby DM, Cornish KM. Linking social behaviour and anxiety to attention to emotional faces in Williams syndrome. *Research in Developmental Disabilities*. 2013;**34**(12):4608-16.
29. Klein-Tasman BP, Albano AM. Intensive, short-term cognitive-behavioral treatment of OCD-like behavior with a young adult with Williams syndrome. *Clinical Case Studies*. 2007;**6**(6):483-92.
30. Klein-Tasman BP, Lira EN, Li-Barber KT, Gallo FJ, Brei NG. Parent and Teacher Perspectives About Problem Behavior in Children With Williams Syndrome. *American Journal on Intellectual and Developmental Disabilities*. 2015;**120**(1):72-86.
31. Lense MD, Dykens EM. Cortisol Reactivity and Performance Abilities in Social Situations in Adults with Williams Syndrome. *American Journal on Intellectual and Developmental Disabilities*. 2013;**118**(5):381-93.
32. Lense MD, Tomarken AJ, Dykens EM. Diurnal cortisol profile in Williams syndrome in novel and familiar settings. *American journal on intellectual and developmental disabilities*. 2013;**118**(3):201-10.

33. Martens M. Developmental and cognitive troubles in Williams syndrome. *Handbook of clinical neurology*. 2013;**111**:291-3.
34. Martens MA, Seyfer DL, Andridge RR, et al. Parent report of antidepressant, anxiolytic, and antipsychotic medication use in individuals with Williams syndrome : Effectiveness and adverse effects. *Research in Developmental Disabilities*. 2012;**33, 6**:2106-21.
35. Meda SA, Pryweller JR, Thornton-Wells TA. Regional Brain Differences in Cortical Thickness, Surface Area and Subcortical Volume in Individuals with Williams Syndrome. *PLoS ONE*. 2012; **7**(2), e31913.
36. Mervis CB, John AE. Cognitive and behavioral characteristics of children with Williams syndrome: Implications for intervention approaches. *American Journal of Medical Genetics, Part C: Seminars in Medical Genetics*. 2010;**154**(2):229-48.
37. Mervis CB, Velleman SL. Children With Williams Syndrome: Language, Cognitive, and Behavioral Characteristics and Their Implications for Intervention. *Perspectives on Language Learning & Education*. 2011;**18**(3):98-107.
38. Mervis CB, Klein-Tasman BP. Williams syndrome: Cognition, personality, and adaptive behavior. *Mental Retardation and Developmental Disabilities Research Reviews*. 2000;**6**(2):148-58.
39. Meyer-Lindenberg A, Hariri AR, Munoz KE, et al. Neural correlates of genetically abnormal social cognition in Williams syndrome. *Nature Neuroscience*. 2005;**8**(8):991-3.
40. Miodrag N, Lense MD, Dykens EM. A Pilot Study of a Mindfulness Intervention for Individuals with Williams Syndrome: Physiological Outcomes. *Mindfulness*. 2013;**4**(2):137-47.
41. Morris CA. The Behavioral Phenotype of Williams Syndrome: A Recognizable Pattern of Neurodevelopment. *Am J Med Genet, Part C: Seminars in Medical Genetics*. 2010;**154C**(4):427-31.
42. Morris CA, Mervis CB. Williams syndrome and related disorders. *Annual Review of Genomics and Human Genetics*. 2000;**1**:461-84.
43. Moskovitz M, Brener D, Faibis S, Peretz B. Medical considerations in dental treatment of children with Williams syndrome. *Oral Surgery Oral Medicine Oral Pathology Oral Radiology and Endodontics*. 2005;**99, 5**:573-80.
44. Munoz KE, Meyer-Lindenberg A, Hariri AR, et al. Abnormalities in neural processing of emotional stimuli in Williams syndrome vary according to social vs. non-social content. *Neuroimage*. 2010;**50**(1):340-6.
45. Ng R, Jaervinen A, Bellugi U. Characterizing associations and dissociations between anxiety, social, and cognitive phenotypes of Williams syndrome. *Research in Developmental Disabilities*. 2014;**35**(10):2403-15.
46. Obrzut JE. Review of Understanding Williams Syndrome: Behavioural Patterns and Interventions. *Child Neuropsychology*. 2004;**10**(4):332-3.
47. Osborne LR, Mervis CB. Rearrangements of the Williams-Beuren syndrome locus: molecular basis and implications for speech and language development. *Expert Reviews in Molecular Medicine*. 2007;**9**(15):1-16.
48. Papaeliou C, Polemikos N, Fryssira E, et al. Behavioural profile and maternal stress in Greek young children with Williams syndrome. *Child Care Health Dev*. 2012;**38**(6):844-53.
49. Perez-Garcia D, Granero R, Gallastegui F, Perez-Jurado LA, Brun-Gasca C. Behavioral features of Williams Beuren syndrome compared to Fragile X syndrome and subjects with intellectual disability without defined etiology. *Research in Developmental Disabilities*. 2011;**32**(2):643-52.

50. Phillips KD, Klein-Tasman BP. Mental health concerns in Williams syndrome: Intervention considerations and illustrations from case examples. *Journal of Mental Health Research in Intellectual Disabilities*. 2009;**2**(2):110-33.
51. Pober BR. Medical progress: Williams-Beuren syndrome. *New England Journal of Medicine*. 2010;**362**(3):239-52.
52. Pober BR, Dykens EM. Williams syndrome: An overview of medical, cognitive, and behavioral features. *Child and Adolescent Psychiatric Clinics of North America*. 1996;**5**(4):929-43.
53. Pober BR, Morris CA. Diagnosis and management of medical problems in adults with Williams-Beuren syndrome. *American Journal of Medical Genetics, Part C: Seminars in Medical Genetics*. 2007;**145**(3):280-90.
54. Riby DM, Hanley M, Kirk H, et al. The Interplay Between Anxiety and Social Functioning in Williams Syndrome. *Journal of Autism and Developmental Disorders*. 2014;**44**(5):1220-9.
55. Rodgers J, Riby DM, Janes E, Connolly B, McConachie H. Anxiety and Repetitive Behaviours in Autism Spectrum Disorders and Williams Syndrome : A Cross-Syndrome Comparison. *Journal of Autism and Developmental Disorders*. 2012;**42, 2**:175-80.
56. Schmitt J. Williams syndrome: Recent developments. *Current Opinion in Psychiatry*. 2001;**14**(5):451-6.
57. Siegel MS, Smith WE. Psychiatric features in children with genetic syndromes: Toward functional phenotypes. *Child and Adolescent Psychiatric Clinics of North America*. 2010;**19**(2):229-61.
58. Smoot L, Zhang H, Klaiman C, Schultz R, Pober B. Medical overview and genetics of Williams-Beuren syndrome. *Progress in Pediatric Cardiology*. 2005;**20**(2):195-205.
59. Stinton C, Tomlinson K, Estes Z. Examining reports of mental health in adults with Williams syndrome. *Research in Developmental Disabilities*. 2012;**33**(1):144-52.
60. Thornton-Wells TA, Avery SN, Blackford JU. Using novel control groups to dissect the amygdala's role in Williams syndrome. *Developmental Cognitive Neuroscience*. 2011;**1**(3):295-304.
61. Udwin O, Yule W. A cognitive and behavioural phenotype in Williams syndrome. *J Clin Exp Neuropsychol*. 1991;**13**(2):232-44.
62. Udwin O, Yule W, Martin N. Cognitive-abilities and behavioral-characteristics of children with idiopathic infantile hypercalcemia. *Journal of Child Psychology and Psychiatry and Allied Disciplines*. 1987;**28**(2):297-309.
63. Urgeles D, Alonso V, Ramos-Moreno T. Neuropsychiatric and behavioral profiles of 2 adults with williams syndrome: response to antidepressant intake. *The primary care companion for CNS disorders*. 2013;**15**(4).
64. Waxler JL, Levine K, Pober BR. Williams syndrome: A multidisciplinary approach to care. *Pediatric Annals*. 2009;**38**(8):456-63.
65. Zarchi O, Attias J, Gothelf D. Auditory and visual processing in Williams syndrome. *The Israel journal of psychiatry and related sciences*. 2010;**47**(2):125-31.

Online Resource B

Supplemental Table 1. Quality Criteria used to rate the studies (adapted from Richards, Jones, Groves, Moss and Oliver 2015)

	Quality Rating			
	**0**<br>**Poor**	**1**<br>**Adequate**	**2**<br>**Good**	**3**<br>**Excellent**
**Sample Identification**	Not specified/reported	Single restricted or non-random sample e.g., a specialist clinic or previous research study[a]<br><br>Single regional sample e.g., a regional parent support group	Multiple restricted or non-random samples e.g., multi-region specialist clinics<br><br>National non-random sampling e.g., national parent support groups	Random or total population sample
**Confirmation of syndrome**[b]	Not confirmed/reported<br><br>Clinical diagnosis only suspected	Clinical diagnosis by ‘generalist’ e.g., General Practitioner or Paediatrician	Clinical diagnosis by ‘expert’ e.g., Clinical Geneticist or Specialist Paediatrician	Genetic confirmation of diagnosis/FISH tested
**Anxiety assessment**	Not specified/reported<br><br>Clinician judgement only	Informant report/self-report instrument e.g., SCAS<br><br>Screening instrument e.g. PAS-ADD<br><br>Clinician judgement against specified diagnostic criteria e.g., DSM-IV or ICD-10	Diagnostic instrument/interviews e.g., K-SADS, ADIS	Consensus from multiple assessments, including at least one diagnostic instrument

[a]For individuals recruited as part of a larger *ongoing* study, if the recruitment strategy is described, it is coded. If not, it is coded as 1, indicating the sample has come from one source (i.e., the larger ongoing study).
[b] Studies can only be classified into a category if all of the participants were tested using the outlined method. For instance, if only 50% of participants were FISH tested, the study cannot receive a score of 3 and will receive a score of 2. For heterogeneous ID studies, a score of 1 is given to studies which include an IQ or adaptive behaviour assessment as part of the study design.

Online Resource C

Statistical Meta-Analytical models

There are various statistical meta-analytical models which can be used to estimate effect sizes. Each model makes different inferences and assumptions regarding the data in question (Hedges and Vevea 2000). The three models referenced in the study are the fixed-effects model, the random-effects model and the quality-effects model. Explanations of the models and justifications for the models used are provided below.

The Fixed Effects (FE) model

The FE model generates effect sizes based on the assumption that studies are homogenous and share common effect sizes (Hedges & Vevea 2000). This model only accounts for within study variability; however it is considered probable that there will also be some level of variation between studies that this model fails to consider. Variability may result from study methodological differences, as well as moderating variables which may act to influence outcomes (Hunter and Schmidt 2000). As a result, Type 1 bias may increase and inaccurate conclusions may be drawn using this model (Field 2003; Overton 1998).

The Random Effects (RE) Model

The RE model accounts for between study variance and is described as providing a more applicable model for real-world data (Borenstein, Hedges, Higgins and Rothstein 2010). The model assumes that effect sizes will vary due to random error and true variation between the studies and redistributes study weightings to account for this (Erez, Bloom and Wells 1996). As a result of the additional sources of variation, the confidence intervals generated in the RE model tend to be larger than those in the FE model (Egger, Smith and Phillips 1997) Even so, the RE model was considered a more appropriate alternative to the FE model for this review, as it considers both within and between study variability.

The Quality Effects (QE) Model

The QE model is a newer method which accounts for methodological differences between studies. This model provides more weight to studies which are of higher quality when estimating effect sizes and this is indicated to be more clinically relevant than the RE model (Doi and Thalib 2008). The redistribution of mathematical weightings corresponds to the parameters ‘0’indicating low quality and ‘1’ indicating high quality (Barendregt, Doi, Lee, Norman and Vos 2013). Quality assessment for this model is required and according to Doi and Thalib (2008), any criteria can be used, providing a Qi score (Quality of the ith score) is derived by dividing individual study quality scores by the maximum score.

The Review

Both a RE model and a QE model were used in this review. These models were chosen for the meta-analysis as they were deemed the most suitable and appropriate for the study’s aims. Both models redistribute mathematical weight to prevent outliers from interfering with the effect size; with the RE model based on statistical heterogeneity and the QE model considering quality (Doi and Thalib, 2008), Usage of both models enabled us to demonstrate the utility of weighting the quality of studies when estimating effect sizes. It also provided some indication as to whether the model’s assumptions had an effect on the prevalence rates estimated.

Models were generated using the statistical package MetaXL 2.0 (Barendregt and Doi 2011).

Online Resource D

Random Effects Forest Plots and Quality Effects Forest Plots for Anxiety Disorders in Williams Syndrome

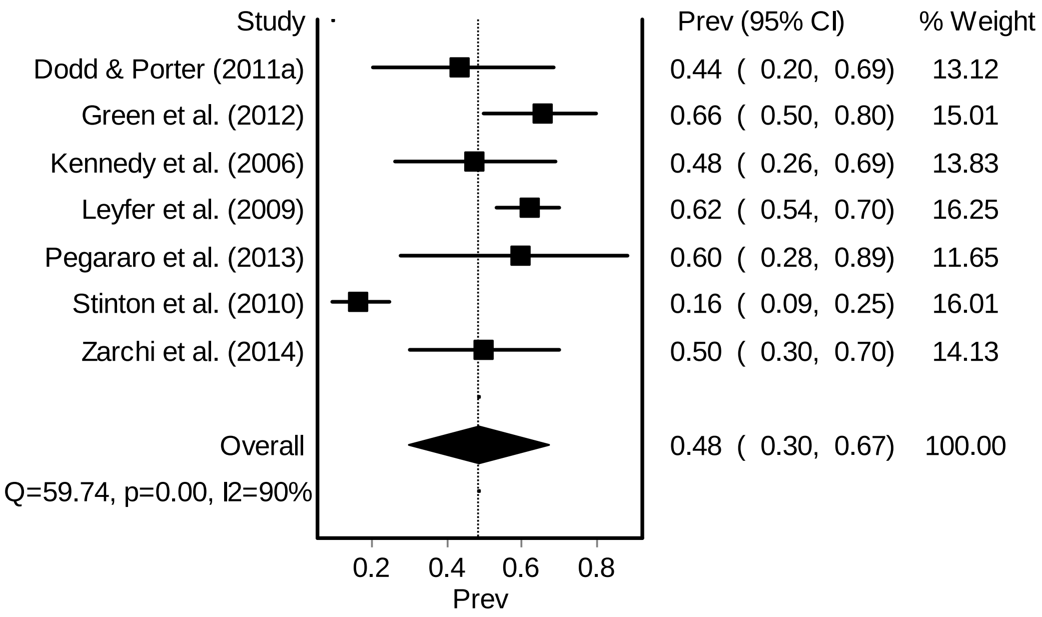

Supplemental Figure 1. Pooled Prevalence estimates for any anxiety disorder using the random effects model.

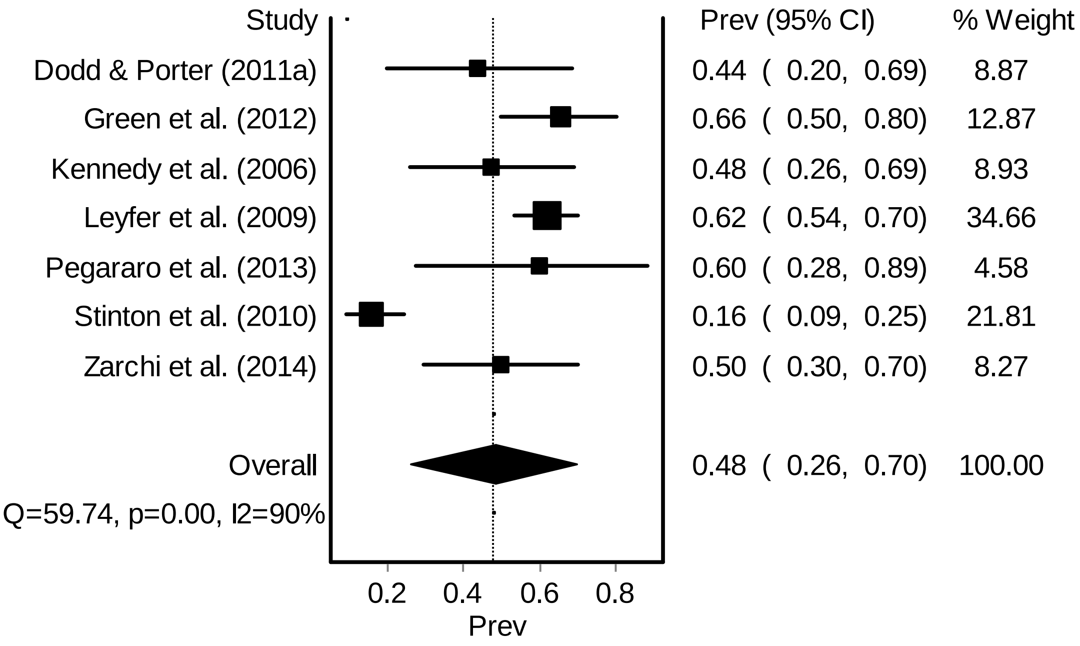

Supplemental Figure 2. Pooled Prevalence estimates for any anxiety disorder using the quality effects model.

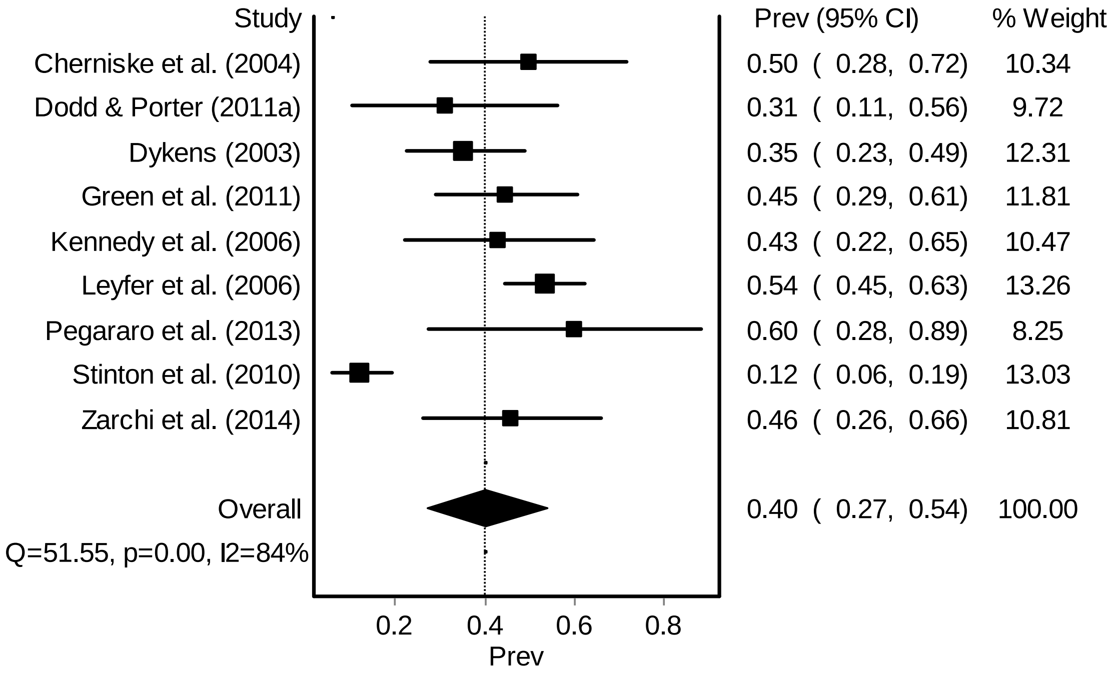

Supplemental Figure 3. Pooled Prevalence estimates for specific phobias using the random effects model.

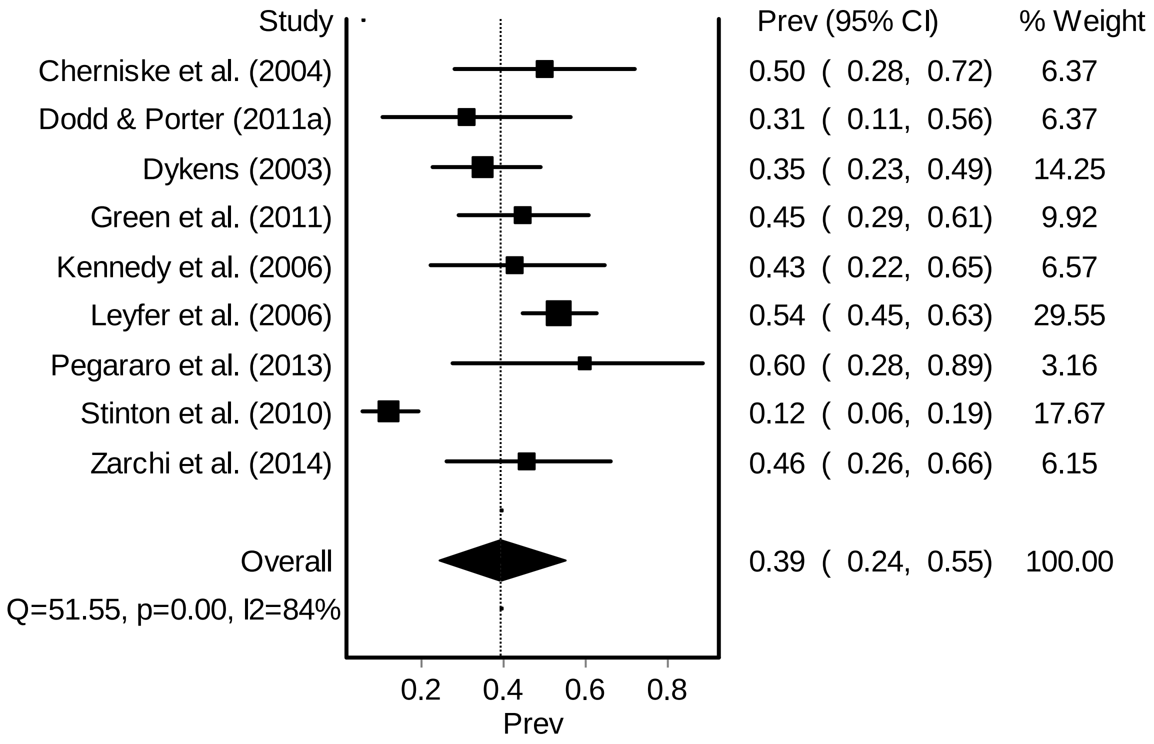

Supplemental Figure 4. Pooled Prevalence estimates for specific phobias using the quality effects model.

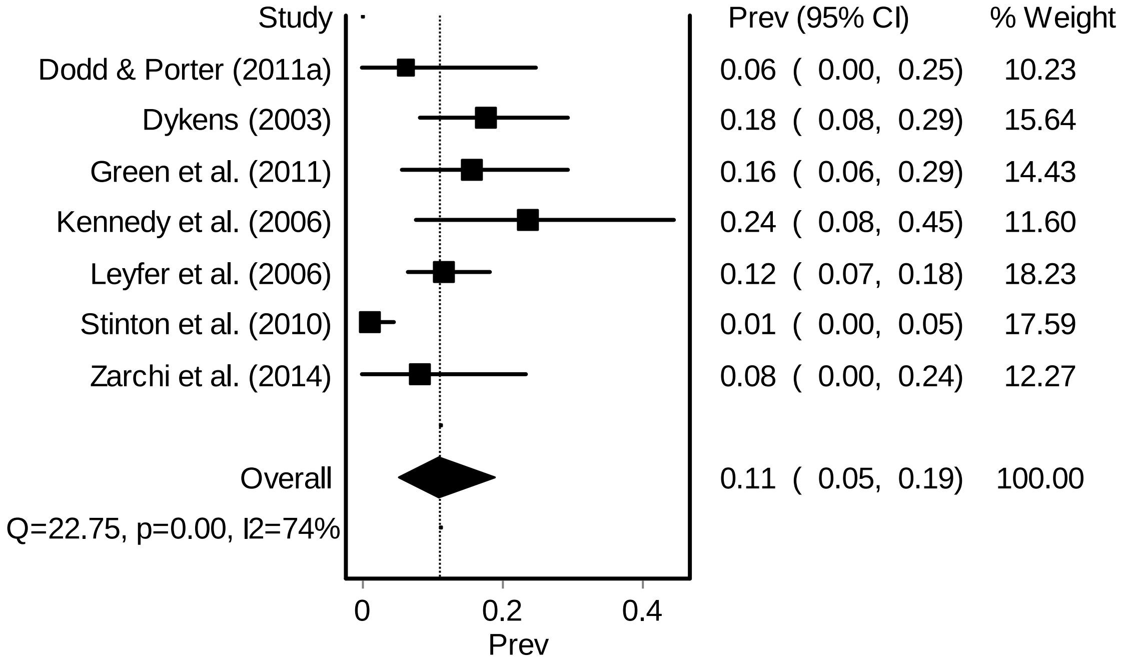

Supplemental Figure 5. Pooled Prevalence estimates for generalised anxiety disorder using the random effects model.

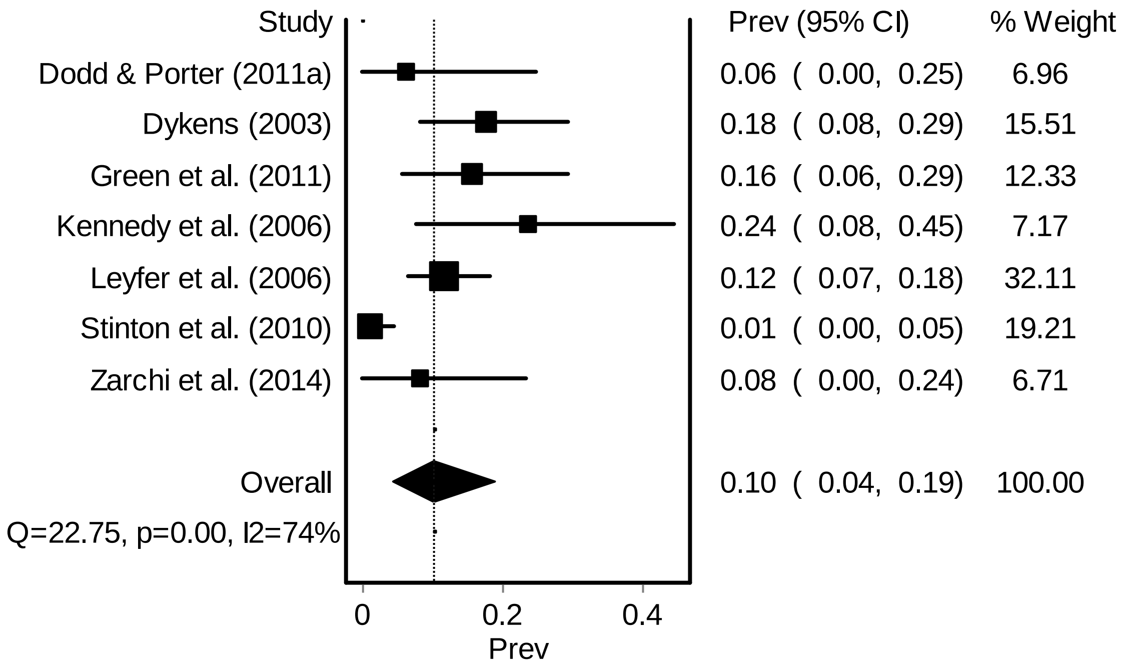

Supplemental Figure 6. Pooled Prevalence estimates for generalised anxiety disorder using the quality effects model.

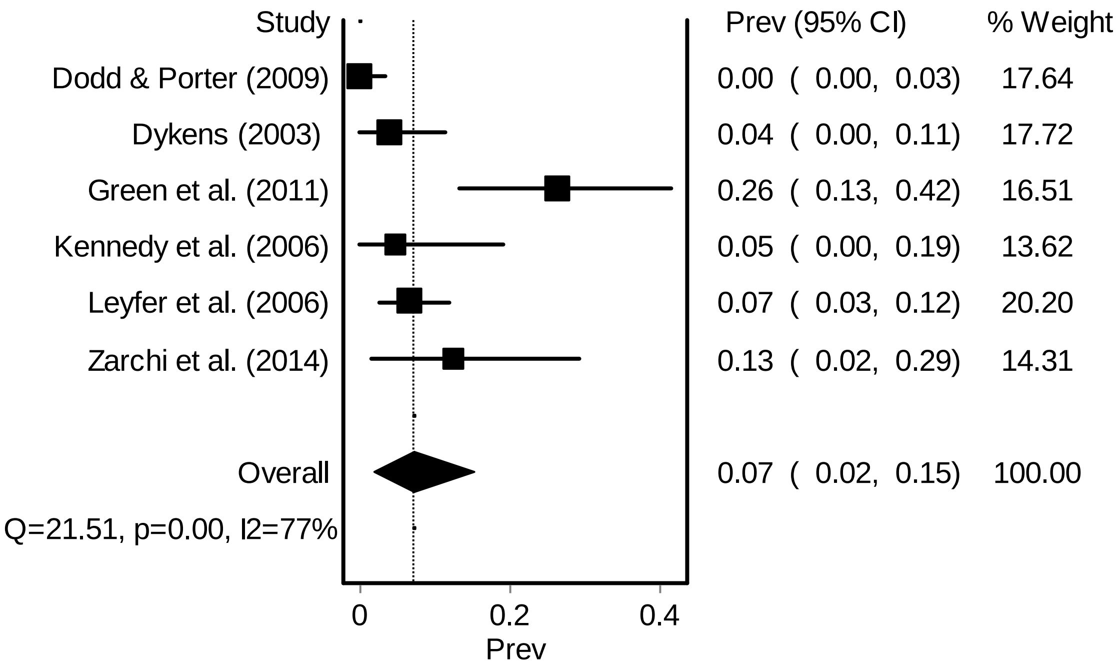

Supplemental Figure 7. Pooled Prevalence estimates for separation anxiety disorder using the random effects model.

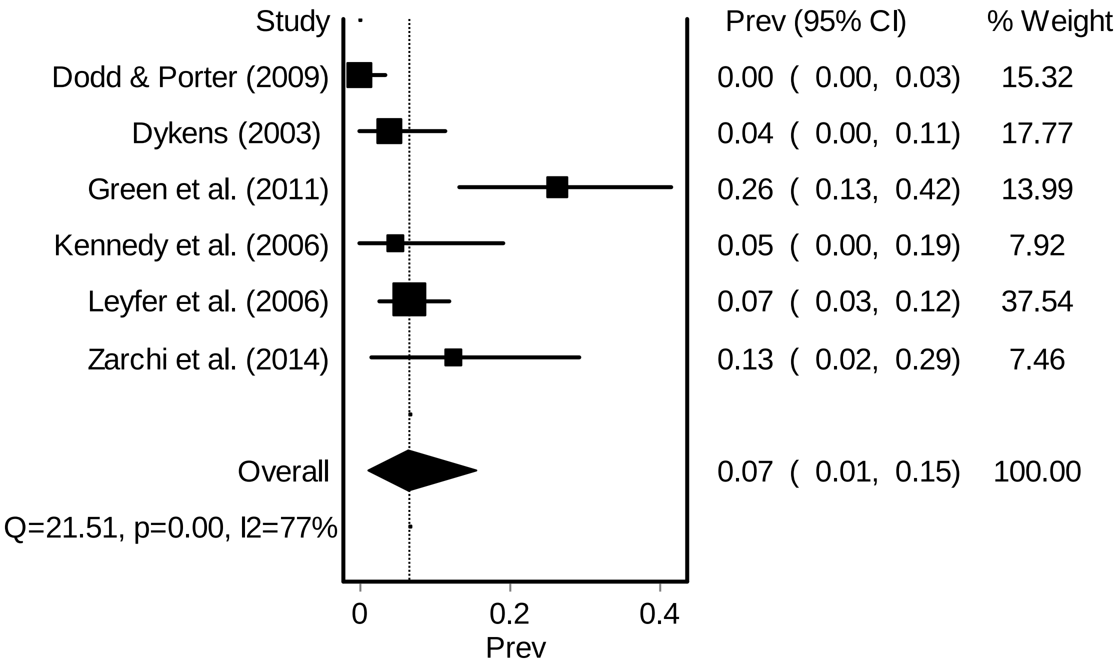

Supplemental Figure 8. Pooled Prevalence estimates for separation anxiety disorder using the quality effects model.

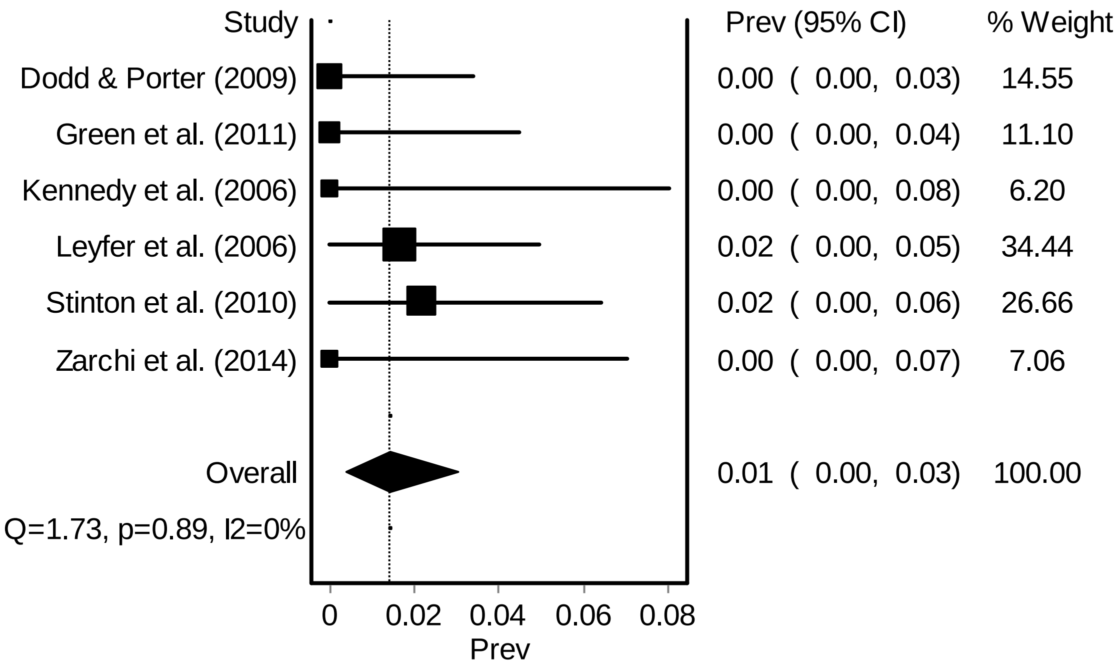

Supplemental Figure 9. Pooled Prevalence estimates for social anxiety disorder using the random effects model.

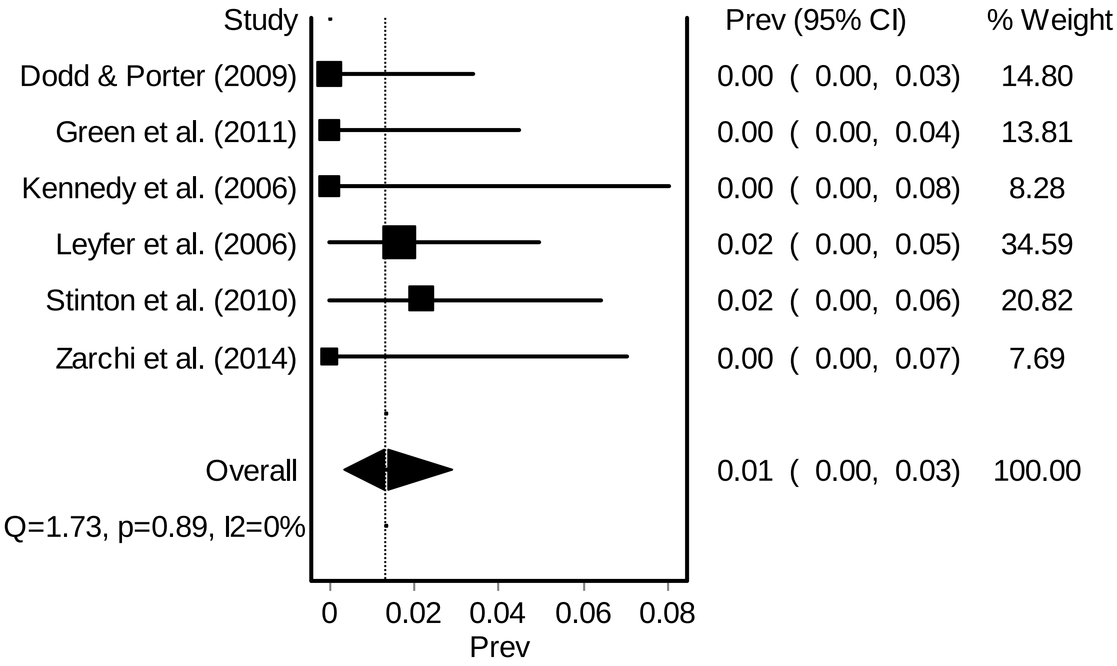

Supplemental Figure 10. Pooled Prevalence estimates for social anxiety disorder using the quality effects model.

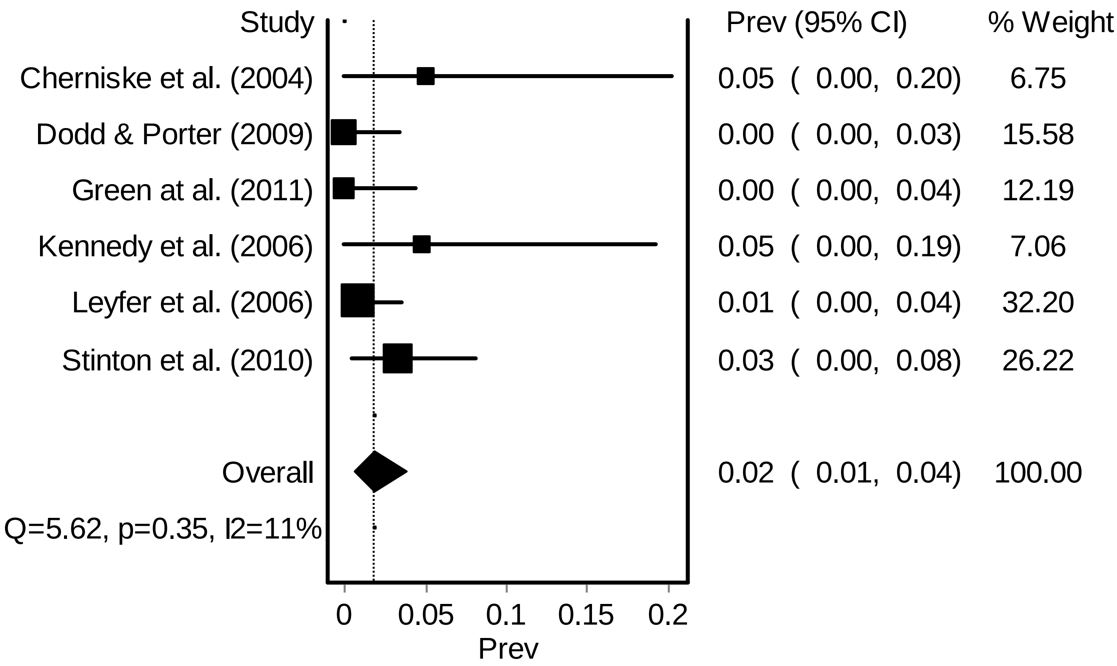

Supplemental Figure 11. Pooled Prevalence estimates for panic disorder using the random effects model.

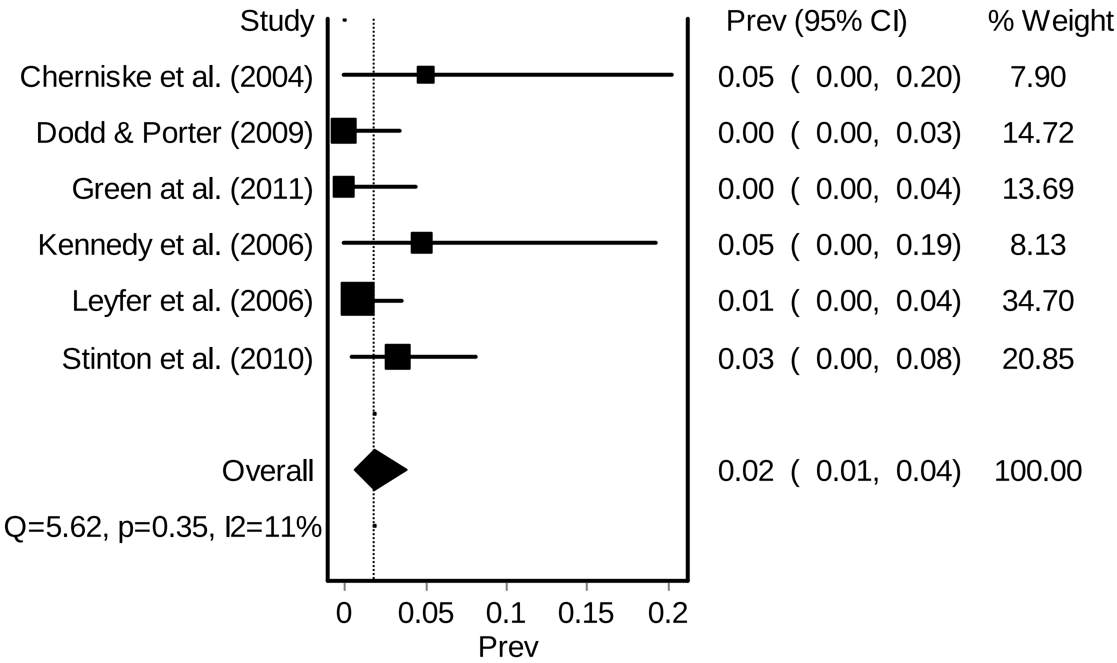

Supplemental Figure 12. Pooled Prevalence estimates for panic disorder using the quality effects model.

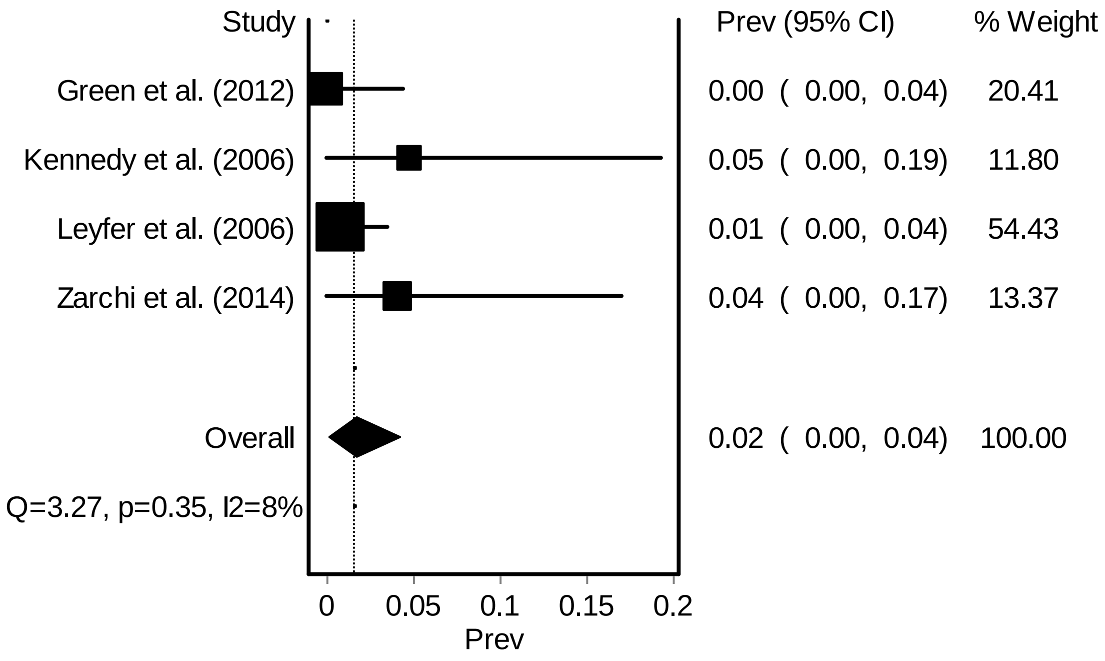

Supplemental Figure 13. Pooled Prevalence estimates for post-traumatic stress disorder using the random effects model.

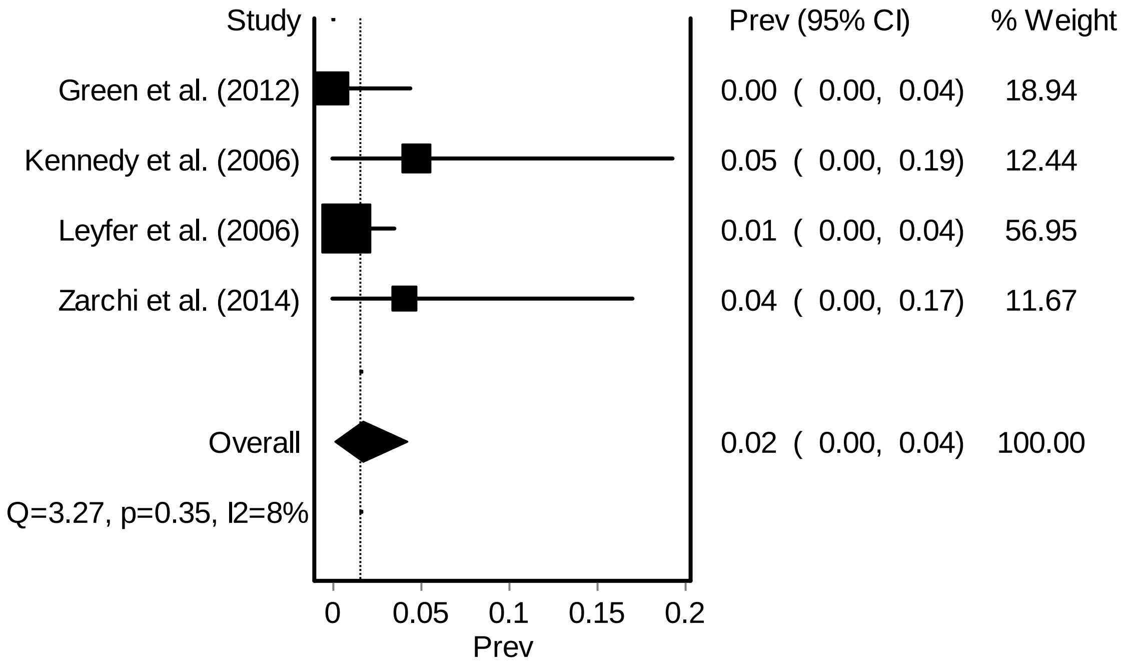

Supplemental Figure 14. Pooled Prevalence estimates for post-traumatic stress disorder using the quality effects model.

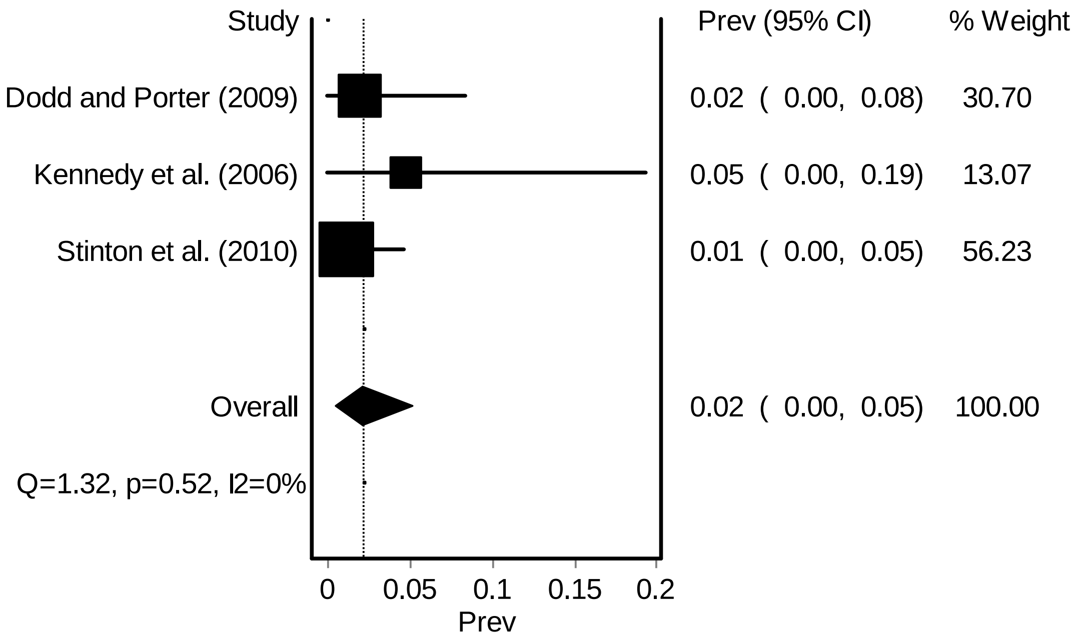

Supplemental Figure 15. Pooled Prevalence estimates for agoraphobia using the random effects model.

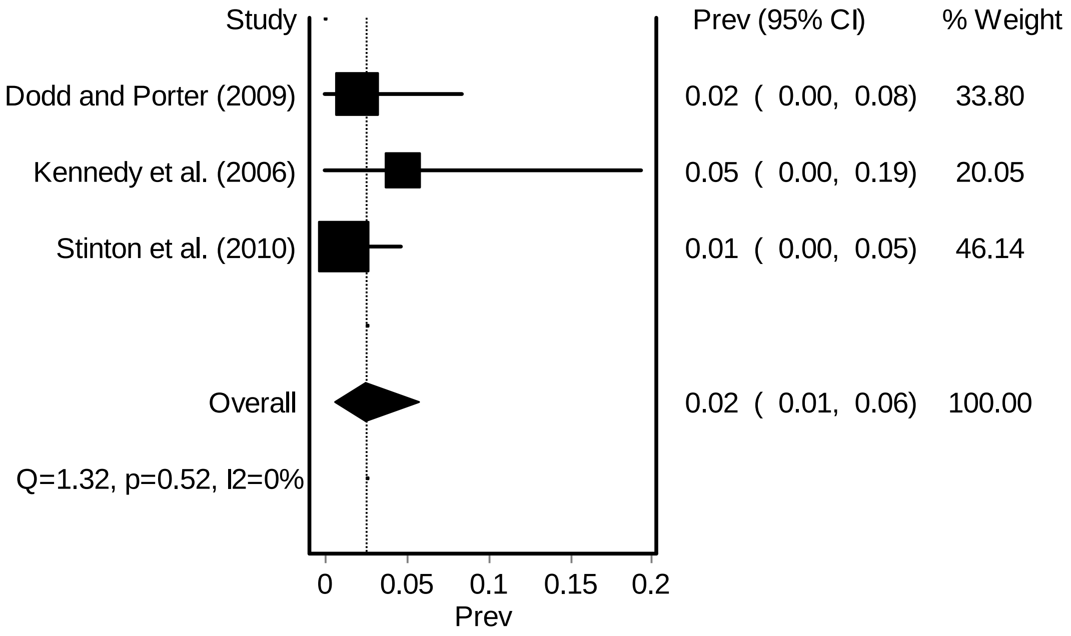

Supplemental Figure 16. Pooled Prevalence estimates for agoraphobia using the quality effects model.

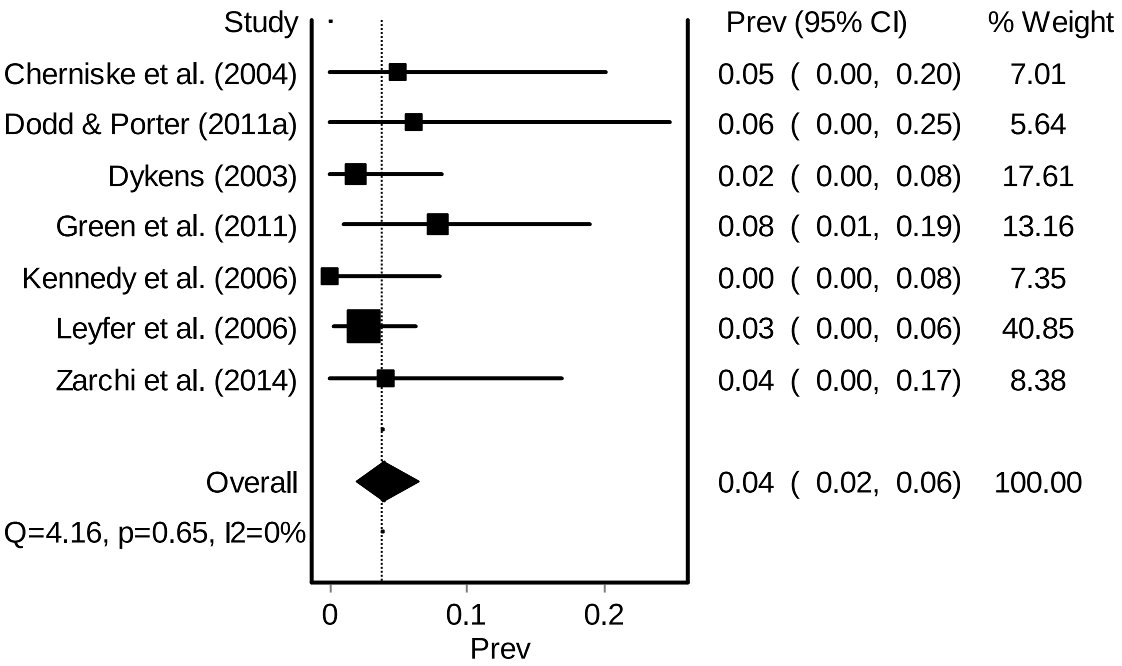

Supplemental Figure 17. Pooled Prevalence estimates for obsessive-compulsive disorder using the random effects model.

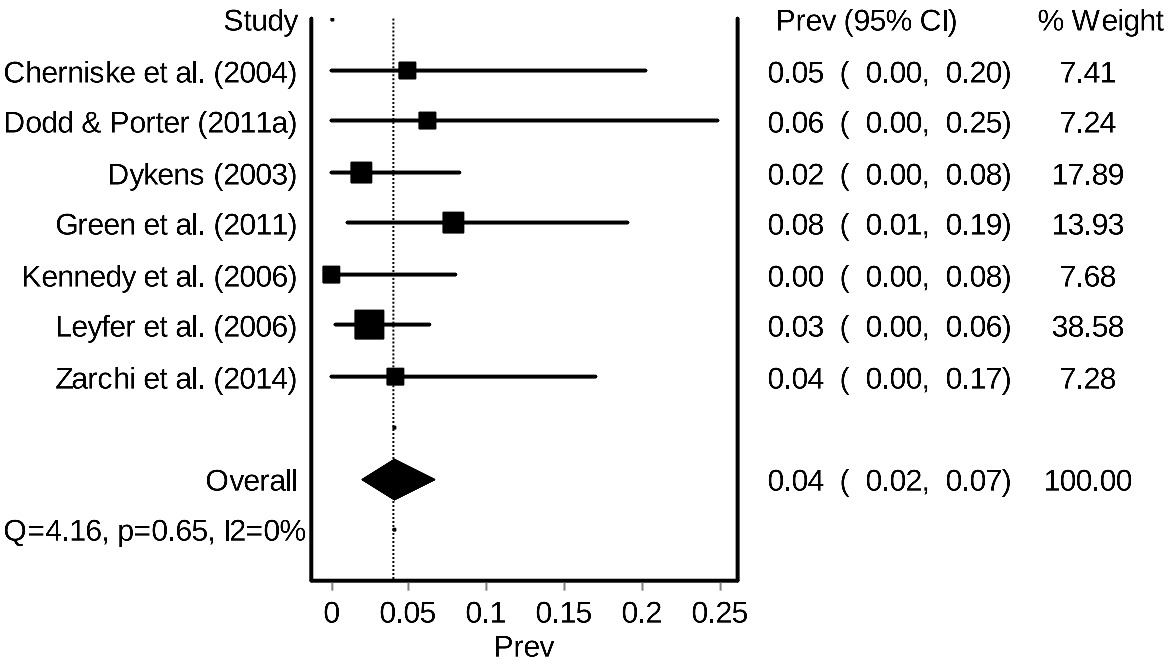

Supplemental Figure 18. Pooled Prevalence estimates for obsessive-compulsive disorder using the quality effects model.

Online Resource E

Pooled prevalence estimates, random effects forest plots and quality effects forest plots for anxiety disorders in individuals with intellectual disability of heterogeneous aetiology

Supplemental Table 2. Total number of included ID studies and participants, mean quality weightings; and random-effects/quality effects models with 95% confidence intervals. Data from Reardon, Gray and Melvin (2015).

	**Included Studies[a]** *(N)*	**Total Ppts** *(N)*	**Mean QW**	**Random- Effects Pooled Prev.** *(CI)*	**Quality- Effects Pooled Prev.** *(CI)*
**Any anxiety disorder**	5	1442	0.49	*10.0 (4.0-18.0)*	*12.0 (5.0-21.0)*
**Specific phobias**	2	1115	0.56	*8.0 (0.0-28.0)*	*7.0 (0.0-26.0)*
**Generalised anxiety disorder**	3	1189	0.56	*1.0 (0.0-3.0)*	*1.0 (0.0-3.0)*
**Separation anxiety disorder**	3	1210	0.56	*4.0 (1.0-9.0)*	*4.0 (1.0-8.0)*
**Social anxiety disorder**	4	1285	0.50	*2.0 (1.0-4.0)*	*2.0 (1.0-4.0)*
**Panic disorder**	2	1115	0.56	*0.0 (0.0-1.0)*	*0.0 (0.0-1.0)*
**Agoraphobia**	2	1115	0.56	*1.0 (0.0-2.0)*	*1.0 (0.0-2.0)*

Notes: QW, Quality Weighting

[a]Overlapping cohorts were removed from each analysis; the overlapping study with the highest quality rating was retained, whilst the others were excluded.

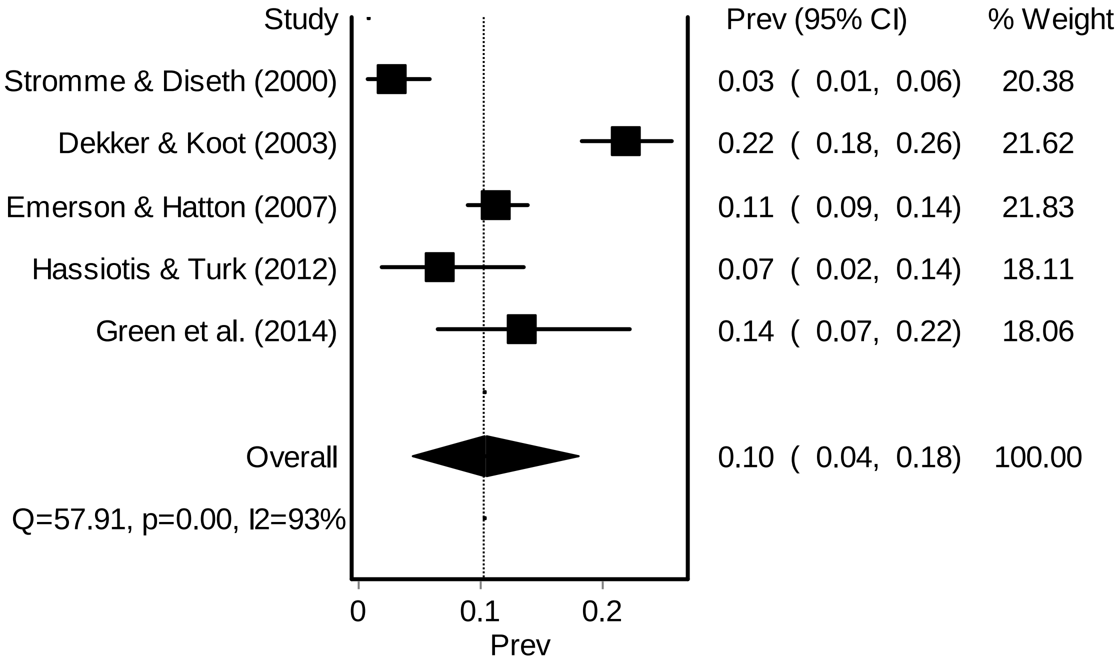

Supplemental Figure 19. Pooled prevalence estimates for any anxiety disorder using the random effects model.

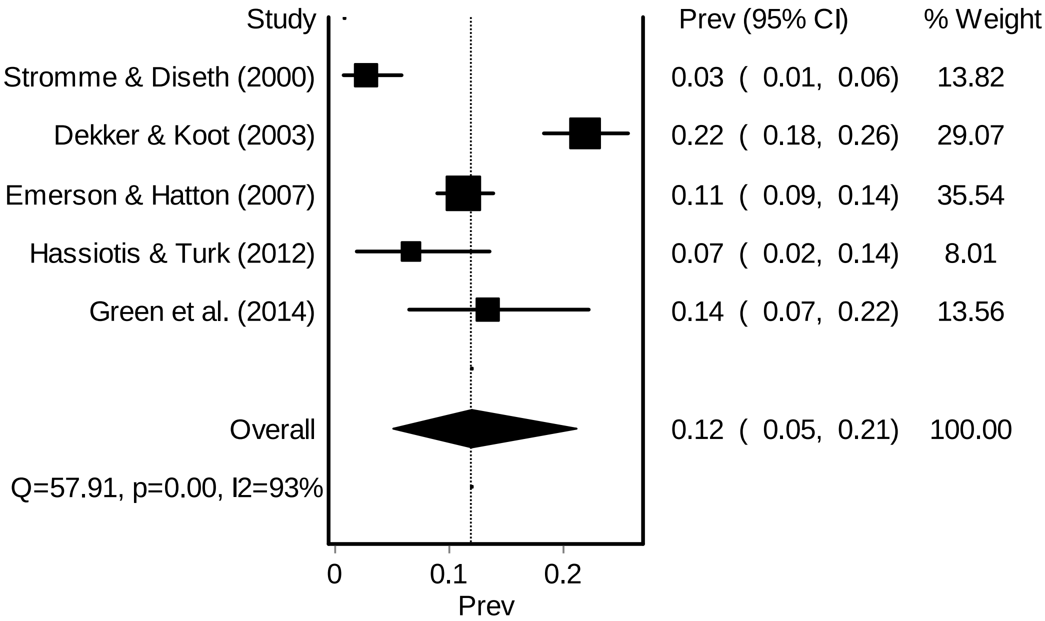

Supplemental Figure 20. Pooled prevalence estimates for any anxiety disorder using the quality effects model.

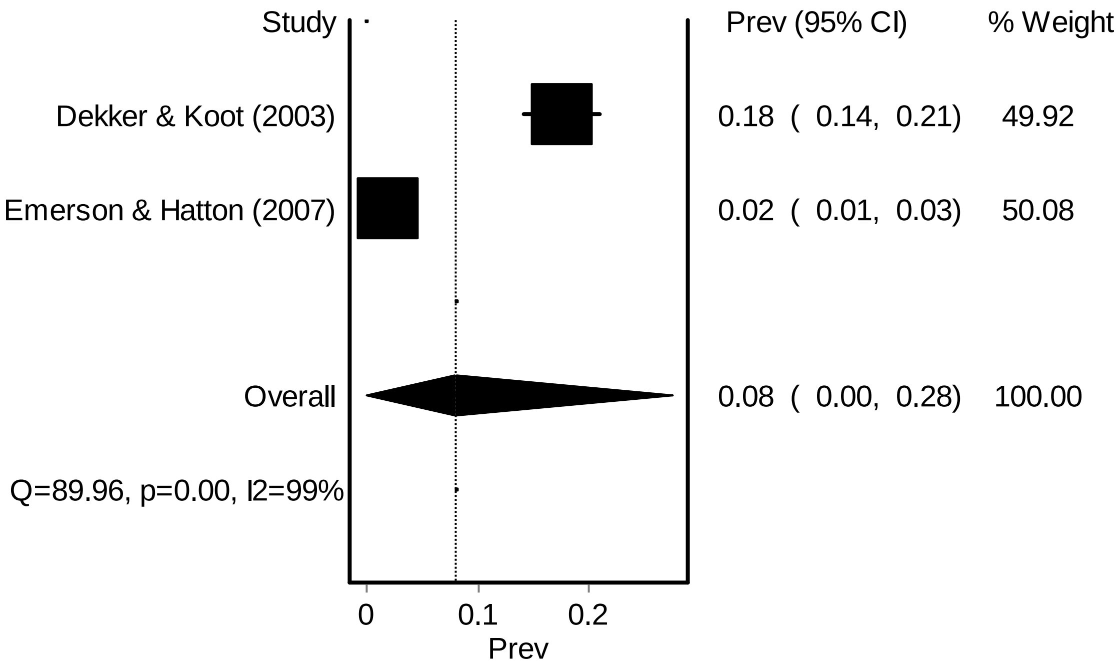

Supplemental Figure 21. Pooled prevalence estimates for specific phobias using the random effects model.

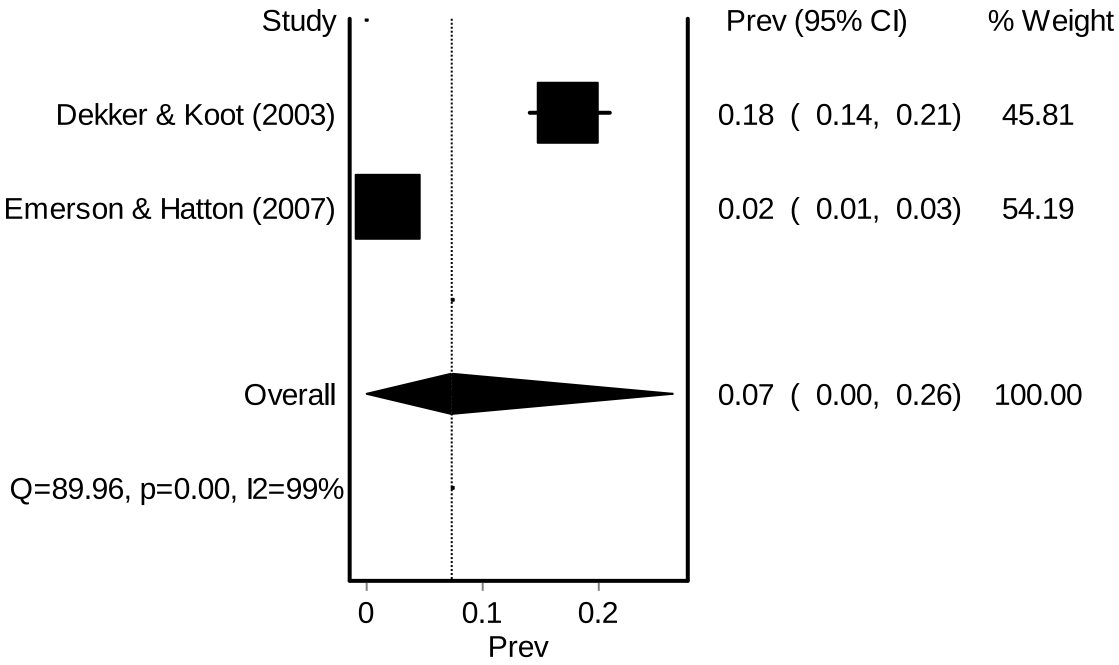

Supplemental Figure 22. Pooled prevalence estimates for specific phobias using the quality effects model.

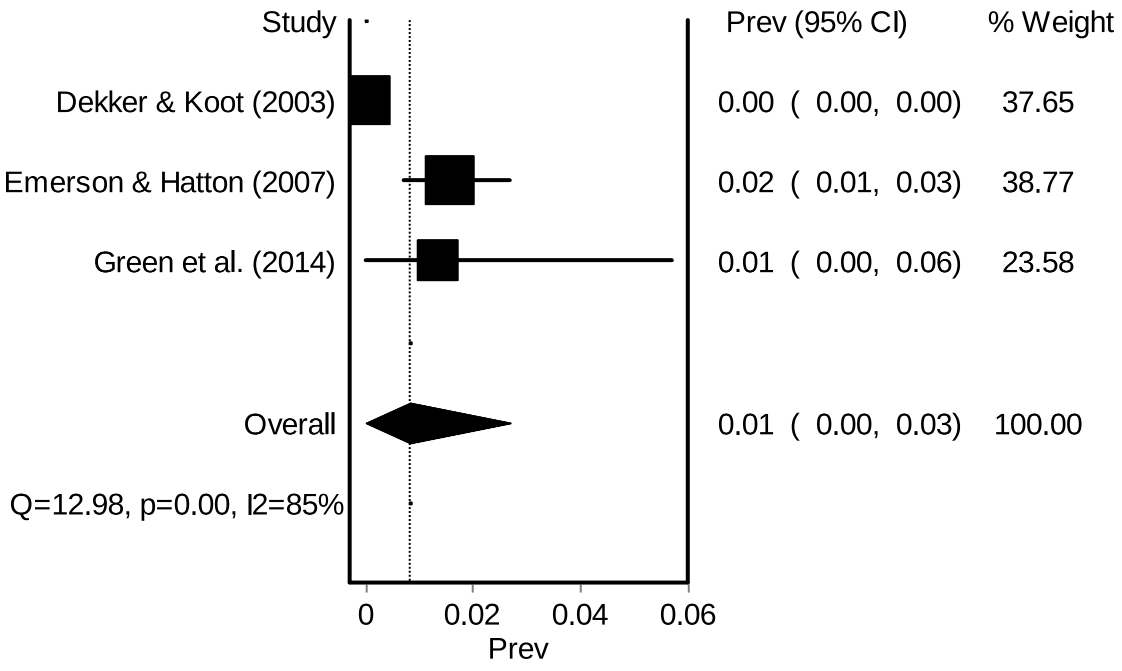

Supplemental Figure 23. Pooled prevalence estimates for generalised anxiety disorder using the random effects model.

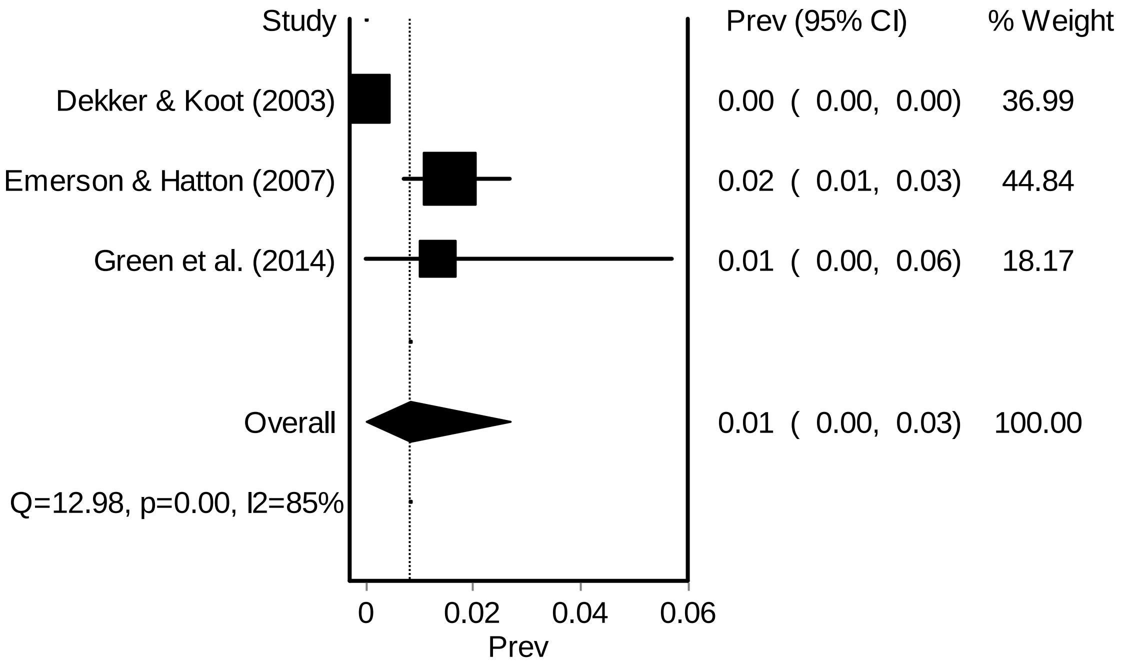

Supplemental Figure 24. Pooled prevalence estimates for generalised anxiety disorder using the quality effects model.

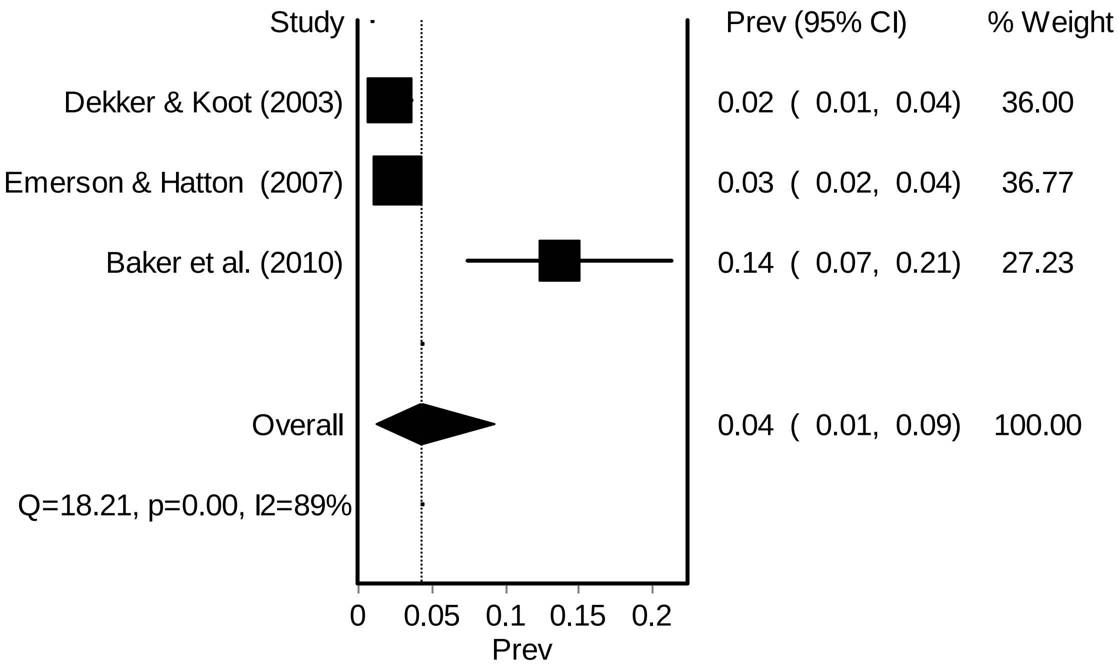

Supplemental Figure 25. Pooled prevalence estimates for separation anxiety disorder using the random effects model.

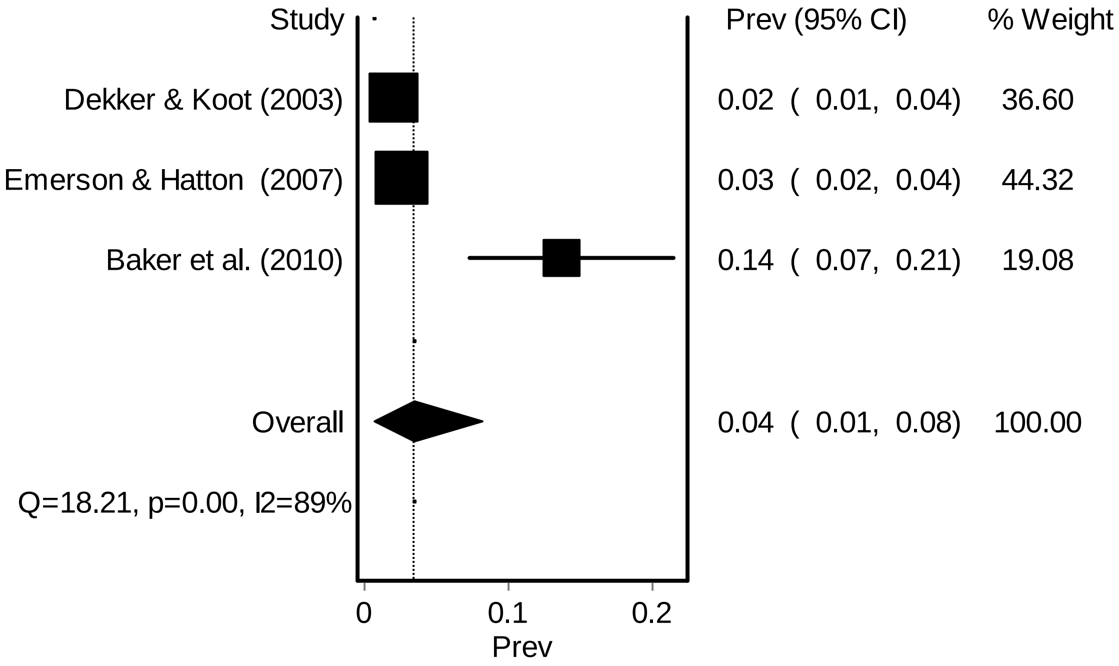

Supplemental Figure 26. Pooled prevalence estimates for separation anxiety disorder using the quality effects model.

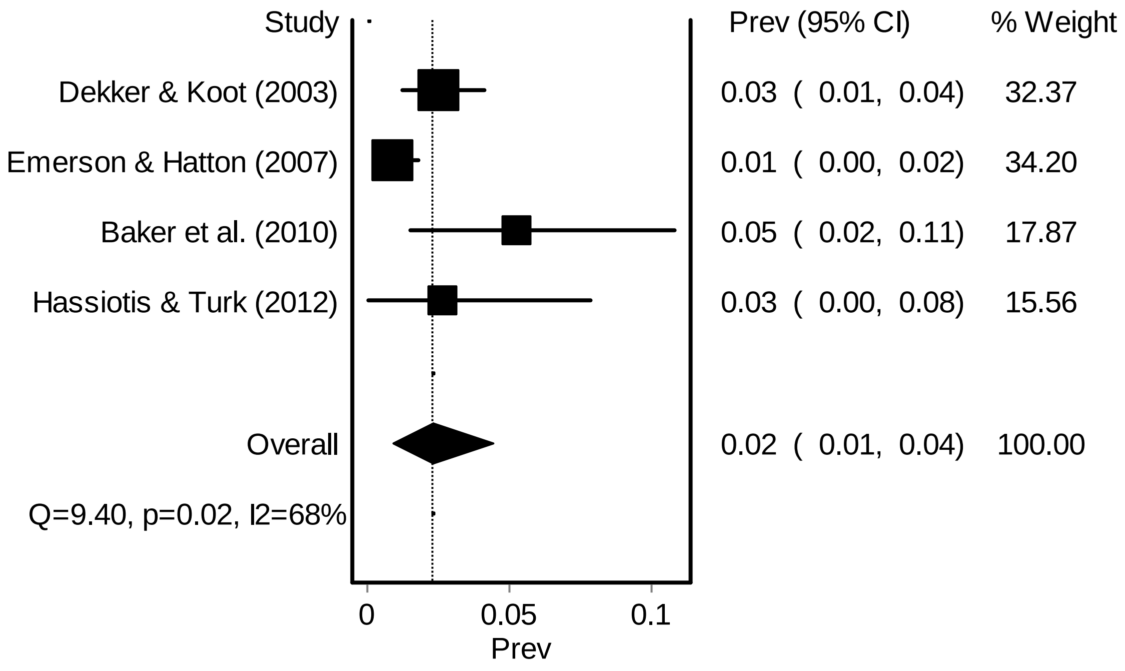

Supplemental Figure 27. Pooled prevalence estimates for social anxiety disorder using the random effects model.

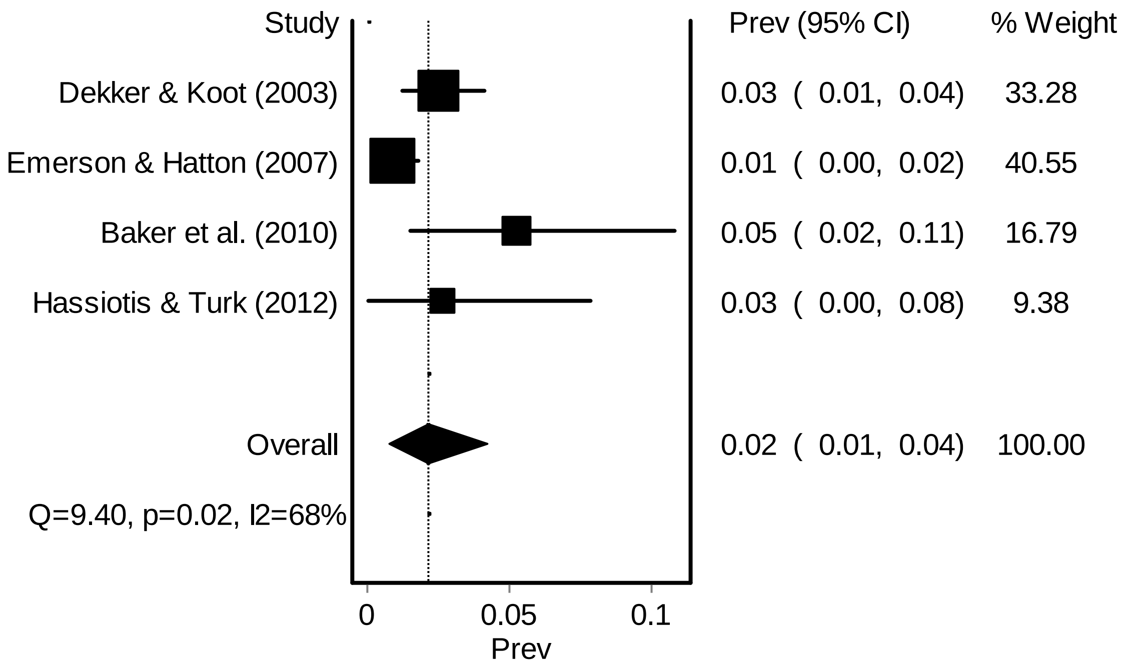

Supplemental Figure 28. Pooled prevalence estimates for social anxiety disorder using the quality effects model.

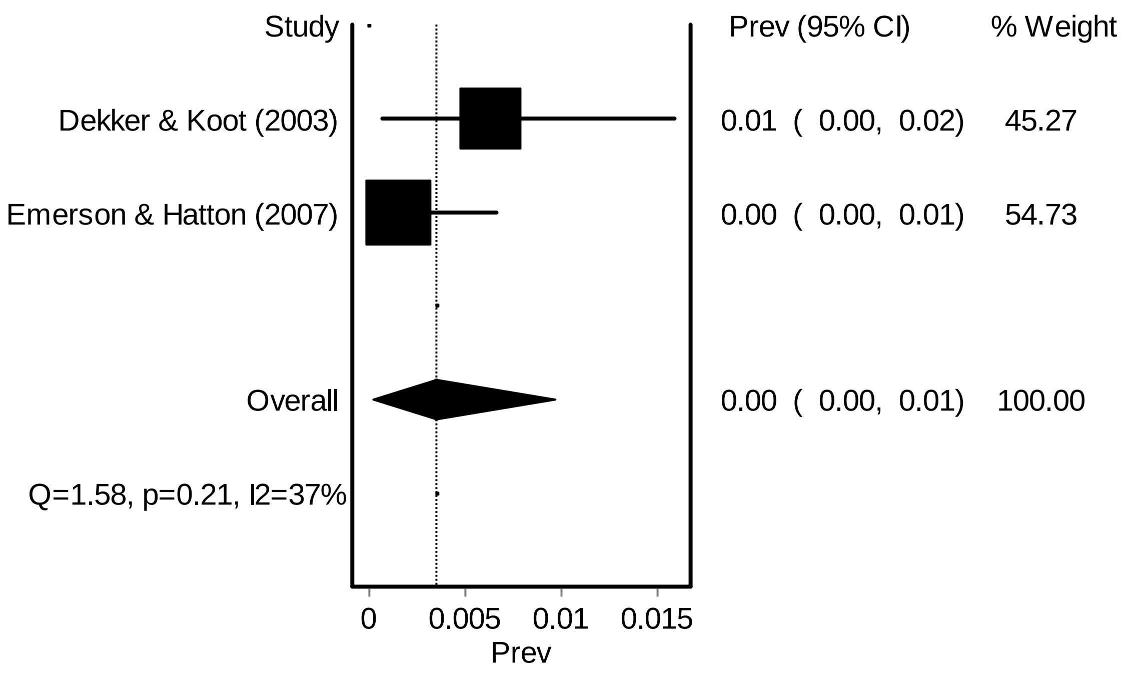

Supplemental Figure 29. Pooled prevalence estimates for panic disorder using the random effects model.

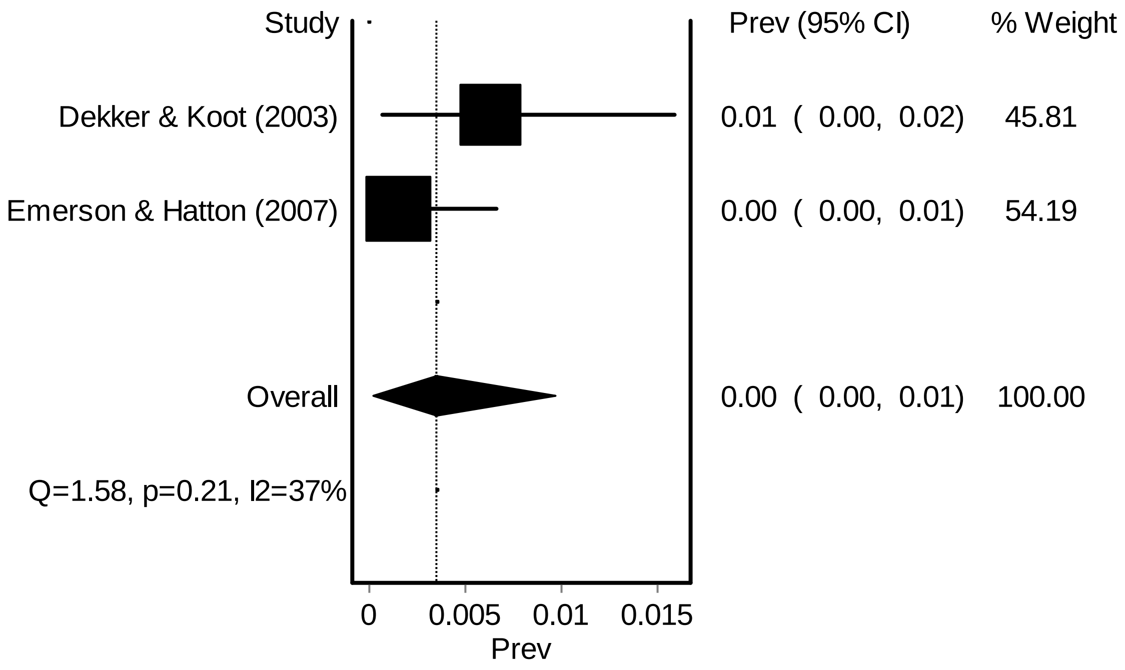

Supplemental Figure 30. Pooled prevalence estimates for panic disorder using the quality effects model.

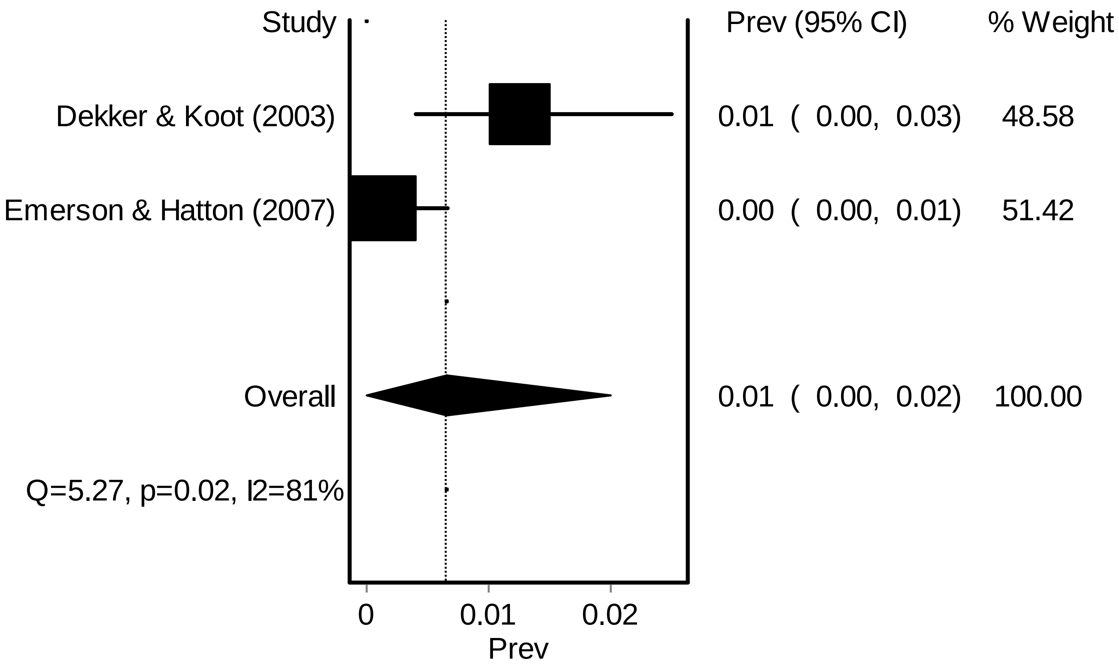

Supplemental Figure 31. Pooled prevalence estimates for agoraphobia using the random effects model.

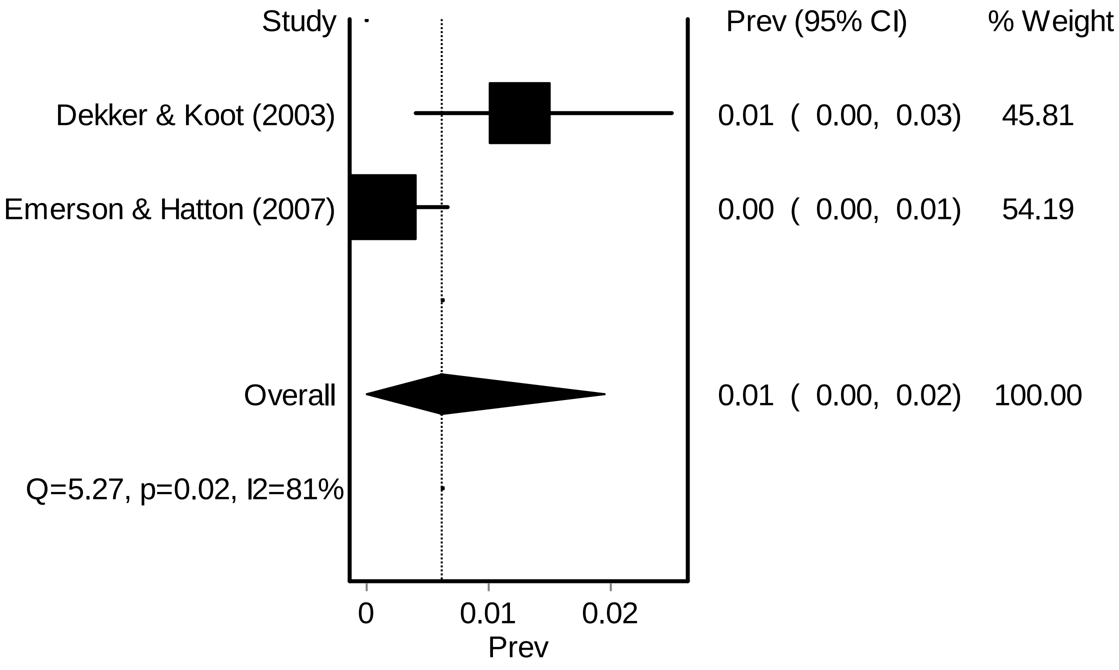

Supplemental Figure 32. Pooled prevalence estimates for agoraphobia using the quality effects model.

Online Resource F

Relative Risk and Odds Ratio statistics

Supplemental Table 3. Relative risk of having an anxiety disorder in individuals with WS compared to individuals with ID (95% confidence intervals).

Type of Disorder	Relative Risk
Any anxiety disorder	**4.00*** (2.27-7.06)**
Specific phobias	**5.57*** (2.62-11.86)**
Generalised anxiety disorder	**10.00* (1.30-76.67)**
Separation anxiety disorder	1.75 (0.53-5.79)
Social anxiety disorder	0.50 (0.05-5.43)
Panic disorder	5.00 (0.24-102.9)
Agoraphobia	2.00 (0.18-21.71)

Notes: ***significant at p<0.0001, *p<0.05

Supplemental Table 4. Odd ratios with 95% confidence intervals of rates of anxiety disorders in WS and ID compared to UK national population estimates (Green, McGinnity, Meltzer, Ford and Goodman 2004).

Type of disorder	Williams Syndrome	Heterogeneous intellectual disability
Any anxiety disorder	**27.05 (8.44-86.74)**	**4.00 (1.14-13.98)**
Specific phobias	**79.28 (8.47-742.13)**	9.33 (0.91-95.97)
Generalised anxiety disorder	**13.78 (1.39-136.75)**	1.25 (0.07-24.01)
Separation anxiety disorder	18.74 (0.76-459.26)	10.38 (0.40-270.90)
Social anxiety disorder	3.36 (0.06-200.45)	6.78 (0.14-317.95)
Panic disorder	10.18 (0.10-1018.2)	0.71 (0.01-83.68)
Agoraphobia	20.39 (0.04-11756.61)	10.09 (0.02-6755.82)
Post-traumatic stress disorder	10.18 (0.10-1018.25)	
Obsessive-compulsive disorder	20.79 (0.23-1870.96)	

Notes: bold=significant

References

Barendregt, J. J., & Doi, S. A. MetaXL User Guide.

Barendregt, J. J., Doi, S. A., Lee, Y. Y., Norman, R. E., & Vos, T. (2013). Meta-analysis of prevalence. *Journal of epidemiology and community health,67*(11), 974-978.

Borenstein, M., Hedges, L. V., Higgins, J., & Rothstein, H. R. (2010). A basic introduction to fixed-effect and random-effects models for meta-analysis. *Research Synthesis Methods*, *1*(2), 97-111.

Doi, S. A., & Thalib, L. (2008). A quality-effects model for meta-analysis. *Epidemiology*, *19*(1), 94-100.

Egger, M., Smith, G. D., & Phillips, A. N. (1997). Meta-analysis: principles and procedures. *BMJ: British Medical Journal*, *315*(7121), 1533.

Erez, A., Bloom, M. C., & Wells, M. T. (1996). Using random rather than fixed effects models in meta-analysis: Implications for situational specificity and validity generalization. *Personnel Psychology*, *49*(2), 275-306.

Field, A. P. (2003). The problems in using fixed-effects models of meta-analysis on real-world data. *Understanding Statistics: Statistical Issues in Psychology, Education, and the Social Sciences*, *2*(2), 105-124.

Green, H., McGinnity, Á., Meltzer, H., Ford, T., & Goodman, R. (2005). Mental health of children and young people in Great Britain, 2004.

Hedges, L. V., & Vevea, J. L. (1998). Fixed-and random-effects models in meta-analysis. *Psychological methods*, *3*(4), 486.

Hunter, J. E., & Schmidt, F. L. (2000). Fixed effects vs. random effects meta-analysis models: implications for cumulative research knowledge. *International Journal of Selection and Assessment*, *8*(4), 275-292.

Overton, R. C. (1998). A comparison of fixed-effects and mixed (random-effects) models for meta-analysis tests of moderator variable effects. *Psychological methods*, *3*(3), 354.

Reardon, T. C., Gray, K. M., & Melvin, G. A. (2015). Anxiety disorders in children and adolescents with intellectual disability: Prevalence and assessment. *Research in developmental disabilities*, *36*, 175-190.

Richards, C., Jones, C., Groves, L., Moss, J., & Oliver, C. (2015). Prevalence of autism spectrum disorder phenomenology in genetic disorders: a systematic review and meta-analysis. *The Lancet Psychiatry*, *2*(10), 909-916.
